# A Systematic Analysis of Hand Movement Functionality: Qualitative Classification and Quantitative Investigation of Hand Grasp Behavior

**DOI:** 10.3389/fnbot.2021.658075

**Published:** 2021-06-07

**Authors:** Yuan Liu, Li Jiang, Hong Liu, Dong Ming

**Affiliations:** ^1^Academy of Medical Engineering and Translational Medicine (AMT), Tianjin University, Tianjin, China; ^2^State Key Laboratory of Robotics and System, Harbin Institute of Technology (HIT), Harbin, China

**Keywords:** hand movement functionality, posture, taxonomies, robotics, human-robot interaction, rehabilitation, human factors

## Abstract

Understanding human hand movement functionality is fundamental in neuroscience, robotics, prosthetics, and rehabilitation. People are used to investigate movement functionality separately from qualitative or quantitative perspectives. However, it is still limited to providing an integral framework from both perspectives in a logical manner. In this paper, we provide a systematic framework to qualitatively classify hand movement functionality, build prehensile taxonomy to explore the general influence factors of human prehension, and accordingly design a behavioral experiment to quantitatively understand the hand grasp. In qualitative analysis, two facts are explicitly proposed: (1) the arm and wrist make a vital contribution to hand movement functionality; (2) the relative position (relative position in this paper is defined as the distance between the center of the human wrist and the object center of gravity) is a general influence factor significantly impacting human prehension. In quantitative analysis, the significant influence of three factors, object shape, size, and relative position, is quantitatively demonstrated. Simultaneously considering the impact of relative position, object shape, and size, the prehensile taxonomy and behavioral experiment results presented here should be more representative and complete to understand human grasp functionality. The systematic framework presented here is general and applicable to other body parts, such as wrist, arm, etc. Finally, many potential applications and the limitations are clarified.

## Introduction

The ability to perform various and skillful tasks using the hand is one of the critical characteristics of humans as a most refined primate, discriminating with other mammals (Cartmill, 1974). In daily life, people may grasp objects securely, such as eating a meal with a knife and fork, perhaps changing the position and orientation of the manipulated objects, such as writing with a pen or touching an object such as an antenna, etc. The human hand is versatile in interactions with the surrounding environment, showing a tremendous functionality (Iberall, 1986). A systematic description of human hand functionality is needed and contributes to the research in many domains around the human hand. In neuroscience, it is not only important to understand the human hand movement behavior, but also to develop the experimental protocol of a cognitive experiment to explore the elaborate mechanisms of motor control (Castiello, 2005). In human–computer interaction in which the hand is used to interact with the computer, specific grasp types selected in advance will serve as a friendly interaction between human and machine (Loclair et al., 2010). In packaging design of necessaries and products on which hands act, it is important to understand the grasp habit during normal use (DiSalvo and Gemperle, 2003). In robotics, understanding hand functionality is essential to mechanical implementation (Catalano et al., 2014; Xiong et al., 2016). In rehabilitation, the extracted critical grasp types can be used to evaluate the remaining capabilities after accidents, disease, or surgery (Light et al., 2002).

However, the complexity of the motor control system makes understanding human hand movement functionality challenging. It is difficult to directly model hand movement functionality from the motor control system. Some key issues are still in the subject of hot debate, such as the brain activity in the grasping process (Sereno and Maunsell, 1998; Castiello, 2005; Goodale, 2010), cortical representations of hand movement–related muscles (Schieber and Hibbard, 1993; Sanes et al., 1995; Meier et al., 2008), biomechanical constraints of hand grasping by tendons and ligaments (Kapandji, 1971; Santello et al., 2013), an accurate kinematic model of the human hand (Stillfried and van der Smagt, 2010; Bullock et al., 2012), etc. In addition, for ethical reasons, substantial investigations about elaborate mechanisms of grasping come from non-human primates (Castiello, 2005), such as macaque monkeys. The substantial differences in hand morphology between human and non-human primates are still in debate (Preuschoft and Chivers, 2012).

Compared with directly modeling the hand movement functionality from the motor control system, hand movement patterns can be seen and measured directly and are also the direct reflections of human hand movement functionality. Meanwhile, for the same given task, movement patterns performed by the motor system are highly stereotyped, between both repetitions and individuals (Wolpert and Ghahramani, 2000). Systematically analyzing hand movement patterns is a feasible way to understand hand movement functionality. As we show the summary of classifying hand movement functionality studies, there is little systematic description of hand movement functionality from both qualitative and quantitative perspectives. There is a lack of a comprehensive view for understanding hand movement functionality systematically.

The contribution of this paper is to provide a systematic framework to help understand hand movement functionality from both qualitative and quantitative perspectives in a logical manner. We qualitatively classify hand movement functionality, build prehensile taxonomy to explore the general influence factors of human prehension, and accordingly design a behavioral experiment to quantitatively understand the way of hand grasp.

In qualitative analysis, we built a hierarchical tree of hand action to classify hand movement functionality to eight action classes. The arm's and wrist's important contributions to hand movement are clarified. Moreover, a prehensile taxonomy containing 52 types is constructed to understand the human prehensile functionality contained stable hold and within hand manipulation in detail. For further exploring the general influence factors of human prehension, the grasp types of prehensile taxonomy are rearranged into object prehensile taxonomy. Two facts are found: (1) the arm and wrist make an important contribution to hand movement functionality; (2) the relative position is a general influence factor significantly impacting human prehension except for the object shape and size. Accordingly, a human grasping experiment is designed to quantitatively understand the way of hand grasping and demonstrate the significant impacts of the general influence factors. Simultaneously considering the impact of relative position, object shape and size, prehensile taxonomy, and the behavioral experiment results presented here should be more representative and complete.

The paper is organized as follows. Section Background presents a systematic discussion of the previous investigations about human hand movement classification. Section Classifying Hand Movement Functionality presents a qualitative analysis of hand movement functionality, including the hand action hierarchical tree and prehensile taxonomy. In section Behavioral Experiment to Investigate Human Grasp Functionality, a behavioral experiment simultaneously considering the impact of relative position, object shape, and size is implemented to quantitatively demonstrate the significant influence and understand the way of hand grasp driven by influence factors. Finally, section Discussion presents some potential uses and discusses the limitations of the presented work.

## Background

In this section, we investigated the previous research about human hand movement classification. Some significant definitions are introduced and arranged in Table 1, which contributes to the later classification. Although substantial efforts have been made to explore hand movements, previous efforts have been mostly made from one particular aspect. To the best of our knowledge, there has been little research on building a systematic framework to understand hand movement functions from both qualitative and quantitative perspectives in a logical manner.

**Table 1:** Some significant definitions of human hand movement.

**Researcher**	**Definition**					
**Schlesinger's classification 1919**	**Object shape**		**Hand surface**			**Hand shape**
	**Cylinder**	**Sphere**	**Tip**	**Palmer**	**Lateral**	**Hook**
	** 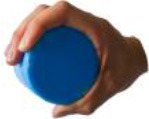 **	** 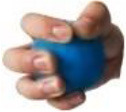 **	** 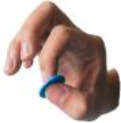 **	** 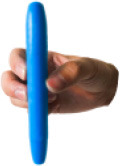 **	** 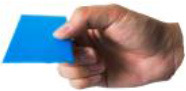 **	** 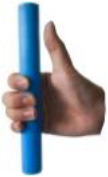 **
Napier (1956)	**Prehensile:** object is seized and held partly or wholly					
	**Non-prehensile:** object is lifted or pushed, and no grasping or seizing is involved					
	**Action goal:** requirements for accomplishing the task					
	**Action goal**	Power	Hold objects stably			
		Precision	Pinch small or large objects, or impart the motion of objects			
Kamakura et al. (1980)	**Contact areas:** the contact area between hand and object while grasping without changing contact					
	**Grip contact areas**	Power	Wide, including a part of the palm, almost entirely on the volar side			
		Intermediate	Including radial aspects of index, middle finger, palm is not included			
		Precision	Between the pulp or tip of the fingers and that of the thumb			
		No thumb	On opposition sides between fingers, thumb is not involved			
Iberall (1986, 1987, 1997)	**Virtual finger (VF):** An abstract representation (a functional unit) for a collection of individual fingers and hand surfaces applying an oppositional force. Real fingers group together into a VF to apply same kind of force or torque opposing other VFs or task torques. **Opposition space:** For a given manual task, this is the area within the coordinates of the hand where opposing forces can be exerted between VF surfaces in effecting a stable grasp.					
	**Opposition**	Palm (a)	Between hand surfaces along a direction perpendicular to the palm.			
		Pad (b)	Between hand surfaces along a direction parallel to the palm.			
		Side (c)	Between hand surfaces along a direction transverse to the palm.			
	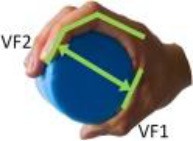		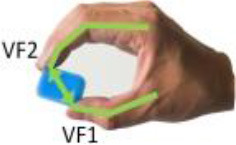		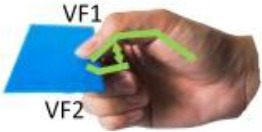	
	(a)		(b)		(c)	
Cutkosky (1989)	**Action goal**		**Object shape**		**VF**	
Feix et al. (2015)	**Action goal**		**Object shape**		**VF**	**Thumb position**
Elliott and Connolly (1984)	**Synergies:** digit movements in common intrinsic manipulative patterns					
	**Synergies**	Simple	All movements of the participating digits are convergent flexion (extension) synergies			
		Reciprocal	Combination movements of the thumb and the other participating fingers show dissimilar or reciprocating movements			
	**Sequential pattern**		Sequenced patterns of movement and synergies for imparting a continuous motion of the object			
Bullock et al. (2013a)	**Motion (e)**		Hand moves with respect to a body coordinate frame			
	**Within hand (f)**		Motion occurs within hand, fingers moves with respect to the hand coordinate frame shown in (d)			
	**Motion at contact (g)**		Hand translates or rotates object with respect to a frame affixed to the contact location(s) on hand			
	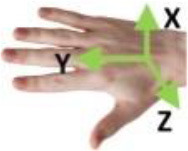		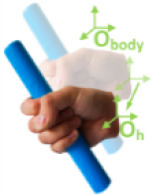		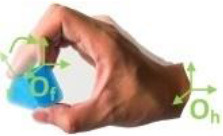	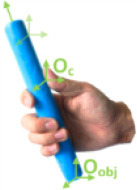
	(d)		(e)		(f)	(g)

Table 1 shows some significant definitions of human hand movement classification. Schlesinger first categorized grasp into six types (see Table 1)—cylindrical, spherical, tip, palmar, lateral, and hook—based on the object shape, hand surfaces, and hand shape (Schlesinger, 1919). Three critical notions (object shape, hand surfaces, and hand shape) were proposed to categorize an enormous variety of grasp types. Napier (1956) divided hand movements into two main groups, prehensile and non-prehensile, based on whether the object is seized and held by the hand or not. For prehensile movement, Napier believes that the grasp can be categorized by task action goals, power, or precision grasps (Napier, 1956), based on whether the task requires large force or precision movement. According to the contact areas on the hand, Kamakura divided the static grasps into four categories (see Table 1): power, intermediate, precision, and no thumb involvement grip categories (Kamakura et al., 1980). Iberall (1986, 1987, 1997) described the hand grasp with a detailed discussion of virtual finger (VF) and opposition spaces (see Table 1). The VF is defined as an abstract representation of fingers applying an oppositional force. Based on the direction of applied force between the hand and object, hand grasps can be divided into three categories: palm, pad and side opposition, which Table 1 shows, recently has been used to analyze the grasp functionality of the bionic hand (Zhan and Liu, 2013). Cutkosky (1989) proposes a hierarchical tree of grasps, which begins with the two basic action goals suggested by Napier and then moves down the tree to VF and object shape, in total listing 16 different grasps (containing one non-prehensile platform type). For exploring human grasping skills in more detail, Feix et al. (2015) constructed a grasp taxonomy containing 33 grasp types. The definitions of action goal, object shape, VF, and thumb position are considered. Feix's GRASP taxonomy is well-structured and has been widely used, such as assisting in determining anthropomorphic hand capabilities (Feix et al., 2012), robotic hand design (Xiong et al., 2016), and experiments analyzing human hand functionality (Juravle et al., 2011; Tessitore et al., 2013). Pollard attempted to refine the previous taxonomies (Abbasi et al., 2016) and used high frame rate handheld cameras to encode for shelf picking and placing actions (Nakamura et al., 2017).

Compared with the previous extensive investigations of human static grasp functionality, there have been relatively few efforts devoted to understanding human manipulative functionality. Elliott and Connolly (1984) believe that, within hand manipulation, movements can be reduced to three basic classes, defined as simple synergies, reciprocal synergies, and sequential patterns (see Table 1). Bullock et al. (2013a) provides a hand-centric classification that contributes to the development of a common framework for describing hand dexterous manipulation; motion within hand and motion at contact are defined as shown in Table 1. Recently, Feix studied the effect of the number of fingers on human precision manipulation workspaces (Feix et al., 2020).

## Classifying Hand Movement Functionality

In this section, hand movement functionality is refined with a logic classification, and then a hierarchical tree of hand action is built for a systematic description of hand movement functionality containing prehension and non-prehension under the consideration of arm and wrist contributions (section Hierarchical Tree of Hand Action), followed by a detailed hand prehensile taxonomy containing 52 distinct grasp types to provide a comprehensive and detailed understanding of human prehensile functionality contained in stable hold and within-hand manipulation (section Hand Prehensile Taxonomy) and finished by decomposing prehensile taxonomy into object prehensile taxonomies to further explore the human grasping general influence factors.

### Analysis of Hand Movement Functionality

**Figure 1:**
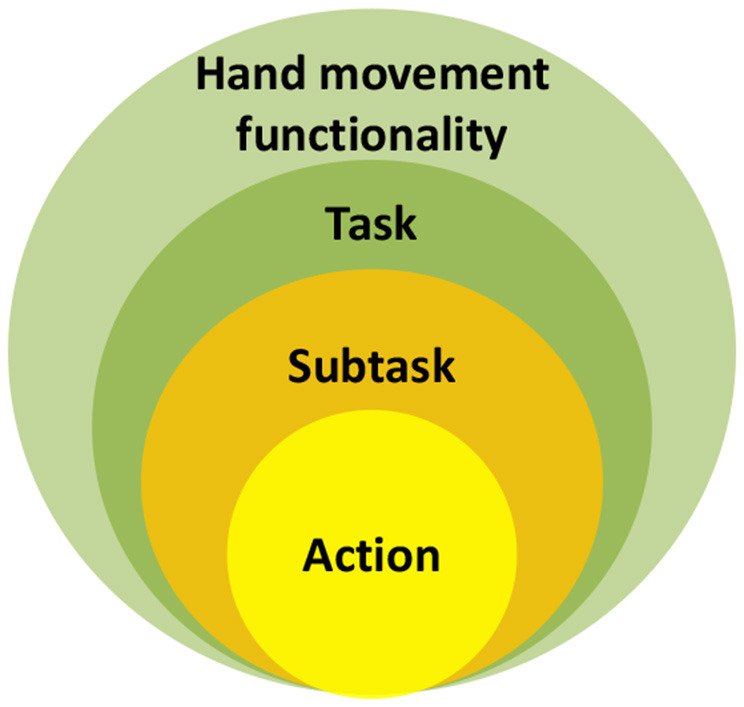
Relationship between hand movement functionality, task, subtask, and action.

Figure 1 presents the logical refining analysis of human hand movement functionality. The relationship between hand movement functionality, task, subtask, and action is provided. The human hand movement functionality is made up of enormous numbers of tasks able to be performed by hand. Each task can be decomposed into subtasks driven by more specific action goals. For accomplishing each subtask, the distinct action should be chosen from the set of possible actions corresponding to the specific action goal. The set of possible actions are termed the action class. Therefore, compared with task and subtask, the action is the smallest composition unit of human movement functionality. Some terms should be clarified:

- **Hand movement functionality**-The sum of the variety of tasks able to be performed by human hands.
- **Task**-Related to human hands, generally specified at a high, often symbolic level, such as drinking water.
- **Subtask**-Decomposed from a task and driven by a more specific action goal.
- **Action class**-Set of possible actions for the specific action goal.
- **Action**-The distinct action chosen from the action class to accomplish the subtask.

Take a simple task such as grasping a cup on the table as an example. This task is one part of hand movement functionality and can be decomposed into two subtasks in sequence: reaching for the cup and grasping it. Whether for reaching or grasping, there are several possible implemented ways that constitute the reaching or grasping action class. While implementing the task, people choose the distinct action from the corresponding action class to implement each subtask in sequence for accomplishing the whole task.

**Figure 2:**
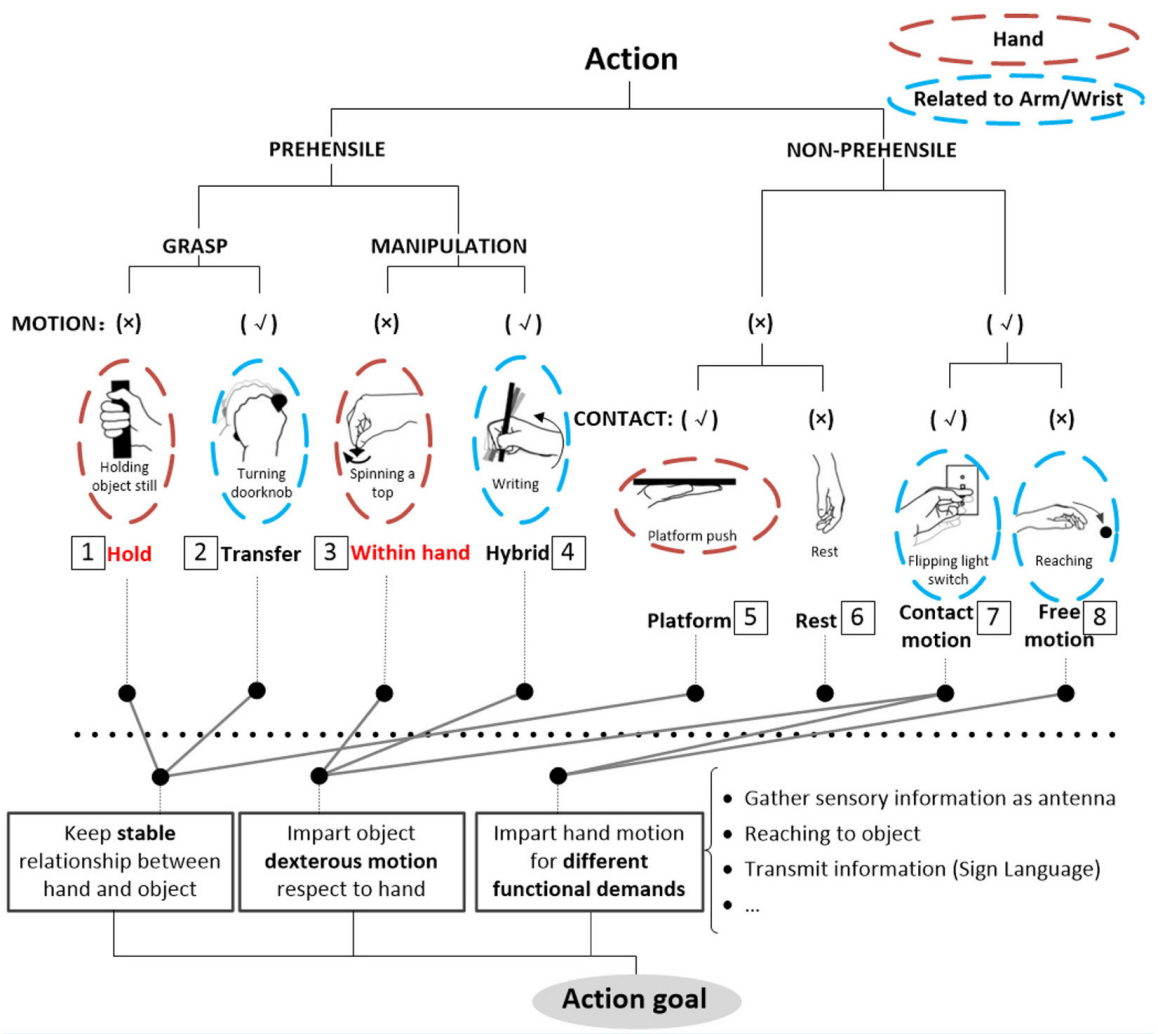
A systematic description of human hand action.

As each action is driven by an action goal, the action goal is important and needs to be clarified. Inspired by Napier's action goals shown in Table 1, we believe the action goals can be summarized as shown in Figure 2: (1) apply forces to keep a stable relationship between hand and object; (2) impart object dexterous motion with respect to the hand, such as transporting an object or in-hand manipulation; and (3) impart hand motion for other functional demands, such as gathering sensory information like an antenna, transmitting information by sign, reaching to an object, etc.

Based on the analysis, we can see that the action of the hand is not only a product of the internal degrees of freedom of the hand, but also the movement of the wrists and arms (Bullock et al., 2013a; Feix et al., 2015). When performing a task using our hands, we reach to objects, grasp and lift them, manipulate them, use them to act on other objects, and finally set them down. In the whole process, the hand, wrist, and arm should be adjusted to various configurations for matching the task requirements. They all need to be considered when describing human hand movement functionality.

### Hierarchical Tree of Hand Action

Figure 2 presents a hierarchical tree to systematically classify human hand actions. Due to the diversity of human hand movements, it is difficult to describe entire hand movements on a very detailed level. To describe them comprehensively and systematically, the action class is set as the smallest unit in the hierarchical tree for classifying the human hand action. For hand prehensile functionality (classes 1 and 3 in Figure 2) merely related to the hand itself, the detailed description is presented in section Hand Prehensile Taxonomy. For a systematic and clear description, the definitions of some terms are clarified and applied in our hierarchical tree.

- **Prehensile**: Object is seized and held partly or wholly, including grasp and manipulation.
- **Nonprehensile:** Object is not seized and held but acts on objects, including a **platform** (class 5) to resist gravity; **contact motion** (class 7), such as flipping a light switch; or **free motion** (class 8) (no objects included) and **rest** (class 6).
- **Grasp:** Keeping a stable relationship between the hand and object, including **hold** (class 1) and **transfer** (class 2).
- **Manipulation:** Motion occurring within the hand is necessary, including **within-hand** [Table 1, as shown in the figure (f) of Table 1] manipulation (class 3) and **hybrid** (class 4), accompanying the finger precision motion and hand motion driven by the wrist and arm [motion definition in Table 1 as shown in figure (e) of Table 1].
- **Motion:** Hand is driven by the wrist and arm and moves with respect to a body coordinate frame as shown in figure (e) of Table 1.
- **Contact:** The hand acts on the object, and there are contact areas between the hand and object.

First of all, one basic choice between *prehensile* and *non-prehensile* actions should be made, starting at the top of Figure 2. For a task in which we need to seize and hold the object partly or wholly, the *prehensile* action should be chosen. Then, the first question is does the object need to be clamped stably (keeping a stable relationship between hand and object) or impart dexterous and precision motion with respect to the hand? If the stable relationship should be kept, then the grasp is chosen; *hold* (class 1) or *transfer* (class 2) is performed according to the task requirement of holding the object stably or transferring object from one place to another. If a dexterous and precision relative motion is needed, then *manipulation* is chosen, and *within-hand* (class 3) or *hybrid* (class 4) manipulation of the object is performed, such as spinning a top using within-hand manipulation or writing using the hybrid manipulation accompanying the finger precision motion and hand motion driven by wrist and arm. For the *non-prehensile* action, the first question concerns whether *contact* between the hand and object is needed? If the task merely needs to support or contact the object, the *platform* (class 5) and *contact motion* (class 7) is performed, such as a platform push, flipping a light switch. If it does not need contact, the action can be divided into *rest* (class 6) and *free motion* (class 8).

Consequently, hand movement action is divided into eight classes. In classification, we find that the contribution of the wrist and arm for hand movement functionality is important. The hand action class related to the arm and wrist is marked by a blue dotted line in Figure 2 to further clarify the arm's and wrist's important role. In particular, the differences between classes 1, 2, 3, and 4 are mainly reflected in whether hand relative motion imparted by the arm and wrist occurs or not. In addition, the arm and wrist movements are also related to the integrity of a task. No matter which kind of prehensile movement, the hand should be transported to the right position to contact the object in the beginning of the task. When the task is finished, the object should be placed at the target location. These actions all need the participation of the arm and wrist and can be described by reaching (class 8) and transferring the object to the end point (class 2). The arm and wrist contribution is also widely reflected in our activities of daily living (ADL), such as sign language to transmit information (class 8), tactile surface exploration to gather sensory information (class 7), etc. Therefore, the arm and wrist are important and make a vital contribution to hand movement functionality.

### Hand Prehensile Taxonomy

**Figure 3:**
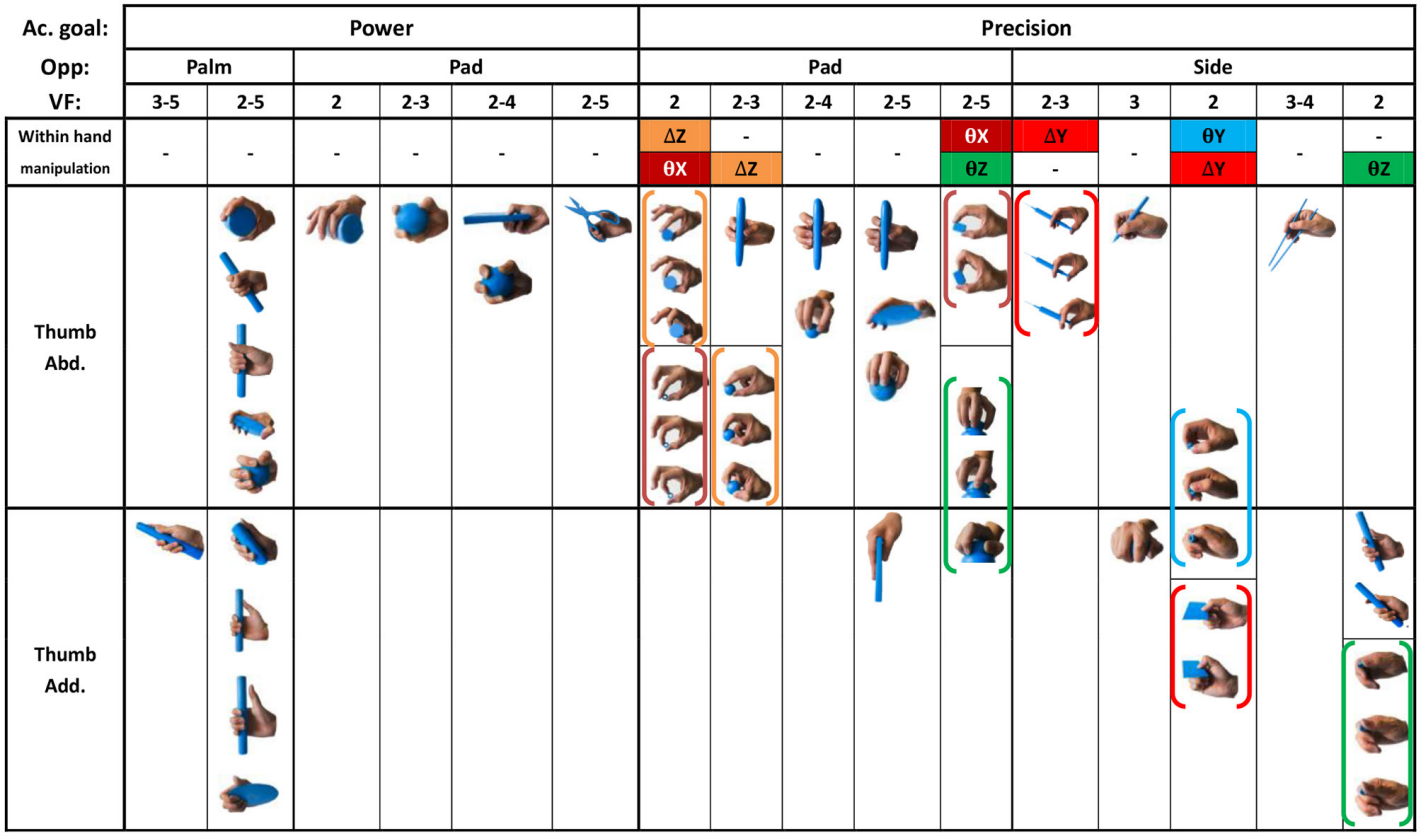
Prehensile taxonomy that incorporates both human grasp and manipulative movements. The grasp types within the each color bracket represent a within hand manipulative movement in sequence.

Figure 3 presents the prehensile taxonomy, providing a detailed summary of hand prehensile function merely related to the hand itself, incorporating hold (class 1) and within-hand manipulation (class 3). The taxonomy contains 52 types. This is the first hand prehensile taxonomy for describing human prehensile functionality containing stable hold and within-hand manipulation from a comprehensive view. To guarantee the comprehensiveness, we compared 29 human hand prehensile investigations, covering neuroscience (Jakobson and Goodale, 1991; Rosenbaum et al., 1996; Santello et al., 1998; Smeets and Brenner, 1999; Cohen and Rosenbaum, 2004; Schieber and Santello, 2004; Ansuini et al., 2008; Bullock et al., 2012; Touvet et al., 2014), hand surgery and rehabilitation (Schlesinger, 1919; Napier, 1956; Kamakura et al., 1980; Light et al., 2002), anatomy (Preuschoft and Chivers, 2012; Santello et al., 2013), biomechanics (Kamper et al., 2003), and robotics (Elliott and Connolly, 1984; Iberall, 1987, 1997; Cutkosky, 1989; Juravle et al., 2011; Feix et al., 2012, 2014a,b, 2015, 2020; Bullock et al., 2013a,b; Tessitore et al., 2013; Zhan and Liu, 2013; Abbasi et al., 2016; Nakamura et al., 2017). For understanding human static grasp ability, Feix provided a good foundation (Feix et al., 2015) and built a grasp taxonomy to cover human static grasping functionality, which has been widely used related to multiple domains. To construct a comprehensive human prehensile taxonomy for covering not only the static hold, but also the within-hand manipulation, we selected two highly cited papers on the classification of within-hand manipulation behavior (Elliott and Connolly, 1984; Bullock et al., 2013a) to help efficiently summarize within-hand manipulation ability in our taxonomy. These two papers classify within-hand manipulation behavior from the view of the manipulative patterns and object motion with respect to the hand coordinate frame, respectively. We unify these two views and arrange them in our prehensile taxonomy. The Electronic Supplementary Material provides a detailed explanation for the selection of the manipulative postures in our taxonomy. Therefore, manipulation behaviors classified in these two representative papers (Elliott and Connolly, 1984; Bullock et al., 2013a) are all covered and presented by finger gaits in our taxonomy.

To provide a systematic and efficient description, 52 types are arranged as shown in Figure 3 based on the terms (action goal, VF, thumb position, opposition, and within-hand) presented in Table 1. For understanding human prehensile behavior or being applied to robotic hand manipulation, it is perhaps the most natural that the within-hand manipulation behavior is classified based on the direction of motion with respect to the hand coordinate frame shown in figure (d) of Table 1. The human hand, radial-ulnar, distal-proximal, and dorsal-palmar axes can be substituted for the x-, y-, and z-axes, respectively. Movements can be described by the motion along hand axes or the combination involving the principal axes: Δz means z-axis translation, ϑz means rotation about the z-axis. Only x-axis translation is not covered as the dexterous motion of x-axis translation (Δx) is difficult for the human hand to implement (Bullock et al., 2013a), which is compared with actual within-hand manipulation functionality. Therefore, as the relative position impact on grasp types are concerned, our taxonomy is expected to provide a more complete understanding of hand prehensile functionality. To further clarify the general influence factors of hand prehension, the impact interference of object (shape and size) is abandoned by dividing the 52 types of prehensile taxonomy to object prehensile taxonomy containing cylinder, sphere, and flat object.

### Object Prehensile Taxonomy

**Figure 4:**
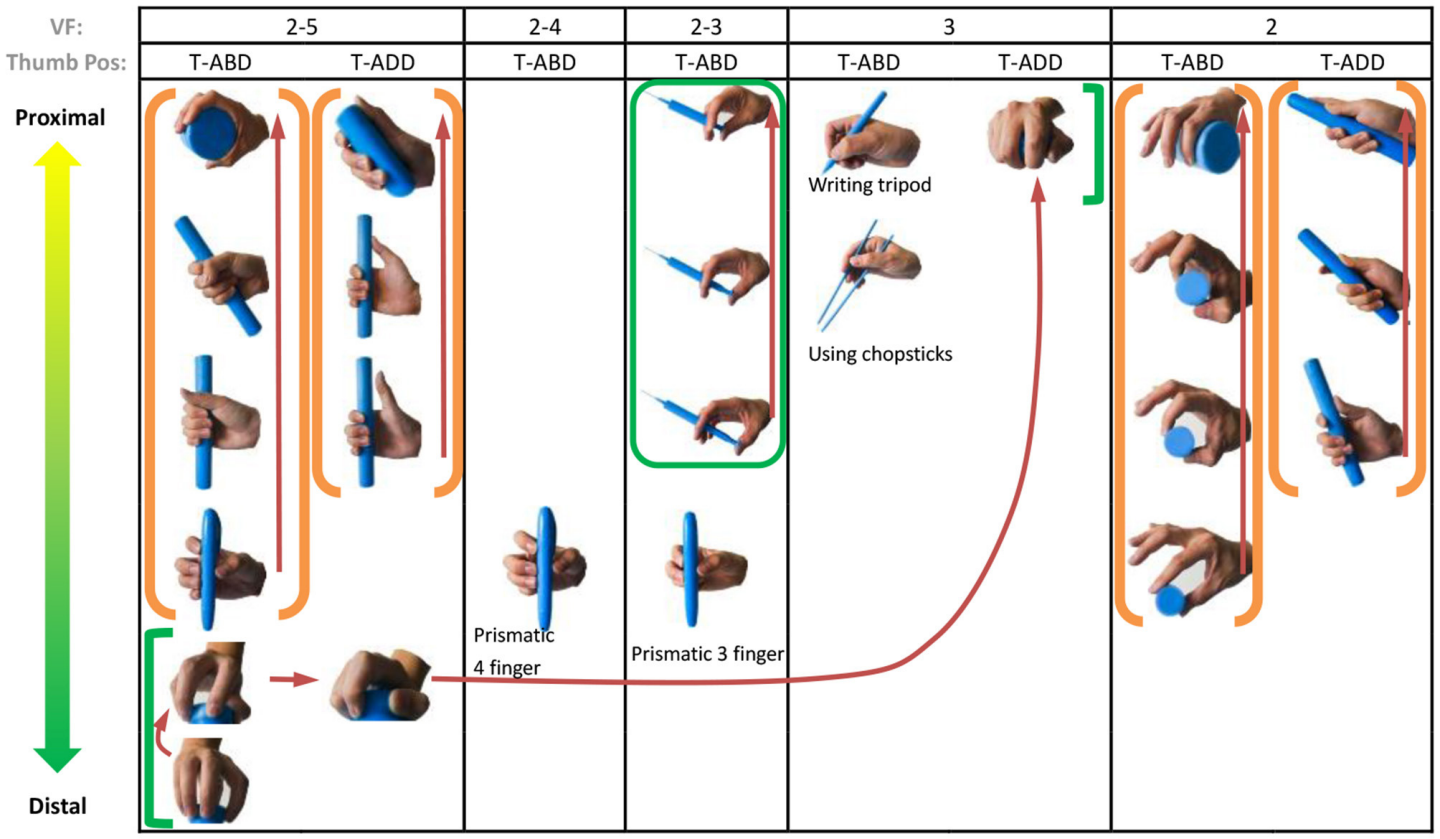
Cylinder object prehensile taxonomy. The grasp types within the golden brackets represent the picking up movement in sequence. The red arrow means the direction of movement execution in sequence. The grasp types within the green box or bracket represent the within-hand manipulation included in Figure 3.

**Figure 5:**
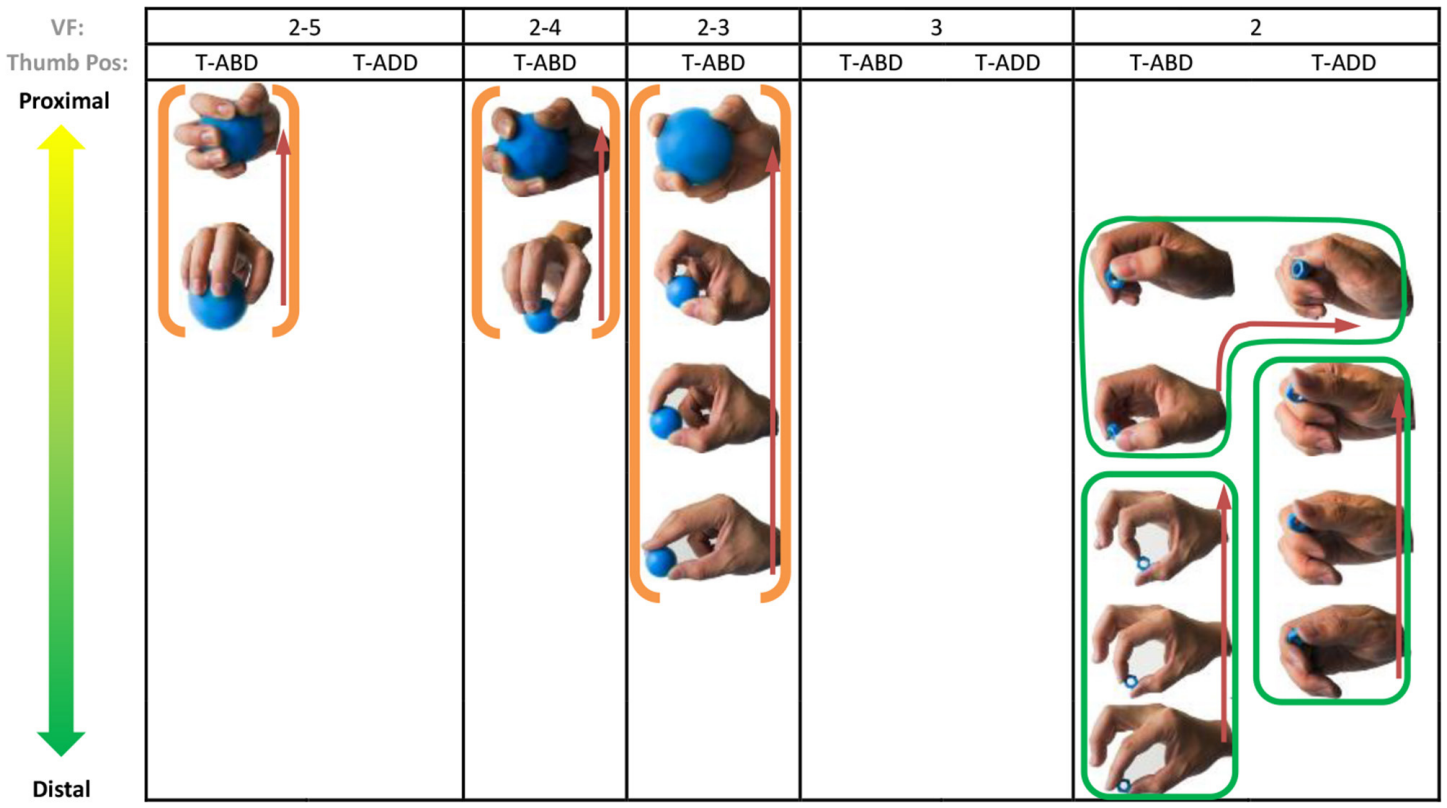
Sphere object prehensile taxonomy. The grasp types within the golden brackets represent the picking up movement in sequence. The red arrow means the direction of movement execution in sequence. The grasp types within the green box or bracket represent the within-hand manipulation included in Figure 3.

**Figure 6:**
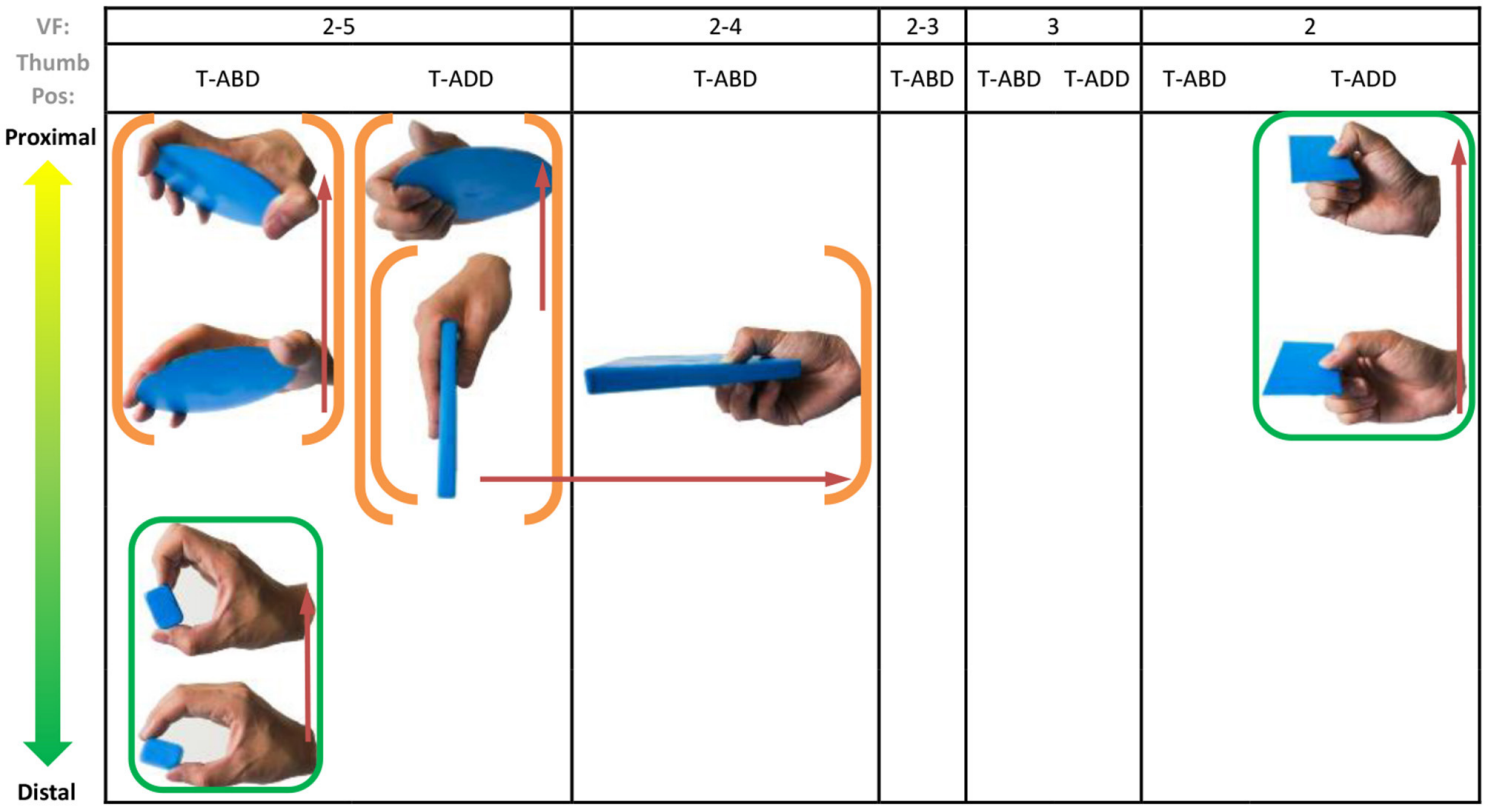
Flat object prehensile taxonomy. The grasp types within the golden brackets represent the picking up movement in sequence. The red arrow means the direction of movement execution in sequence. The grasp types within the green box or bracket represent the within-hand manipulation included in Figure 3.

All 52 prehensile types in Figure 3, except for the using scissor type, are rearranged into cylinder, sphere, and flat object prehensile taxonomy (Figures 4–6) based on the object shape, respectively. Many grasps have similar VF; therefore, some cells are populated with more than one grasp. Within each cell and for the same thumb position, the difference between the grasps is mainly the relative position between object and human hand. In this case, the grasps can be sorted according to the relative distance from distal to proximal as shown in Figures 4–6. Then, a striking observation is that both precision and power grasps can be sorted into within-hand manipulation tasks, mostly showing an object pickup process from distal to proximal to the palm.

From Figures 4–6, we can observe the following:

1) The relative positions significantly impact human prehension. The relative position between the human hand and objects is an important factor leading to the diversity of prehensile types. As shown in Figures 4–6, the grasp types in the same golden brackets, green boxes, and brackets vary significantly because of the impact of changing relative position and orientation between hand and objects.
2) The complex and diverse human precision and power grasp types mostly can be concisely described by human pickup motion. Human precision and power grasp types can be both arranged and reflected in the object pickup sequential process from distal position to the palm as shown in the golden brackets of Figures 4–6. This kind of pickup behavior is commonly used in our daily life, especially for grasping a small object. For instance, human hands pick up a thin stick on the table (shown as the prehensile types within the left upper golden brackets of Figure 4). At the beginning, the precision grasps are used to pinch the stick in the distal position. Subsequently, the stick is pressed to the palm by flexing the finger proximal interphalangeal (PIP) joints. Hereafter, finger MCP joints largely flex, and then the stick is stably grasped. By now, the object is picked up to the palm and the whole pickup motion is finished.

## Behavioral Experiment to Investigate Human Grasp Functionality

According to the observation obtained from object prehensile taxonomy analysis, we know that, except object (shape and size), relative position significantly impacts human prehension. Therefore, after simultaneously considering the impact of relative position, object shape, and size, we designed a laboratory-based unstructured behavioral experiment to comprehensively understand the human grasp and qualitatively demonstrate the significant impact factors on human prehension.

**Figure 7:**
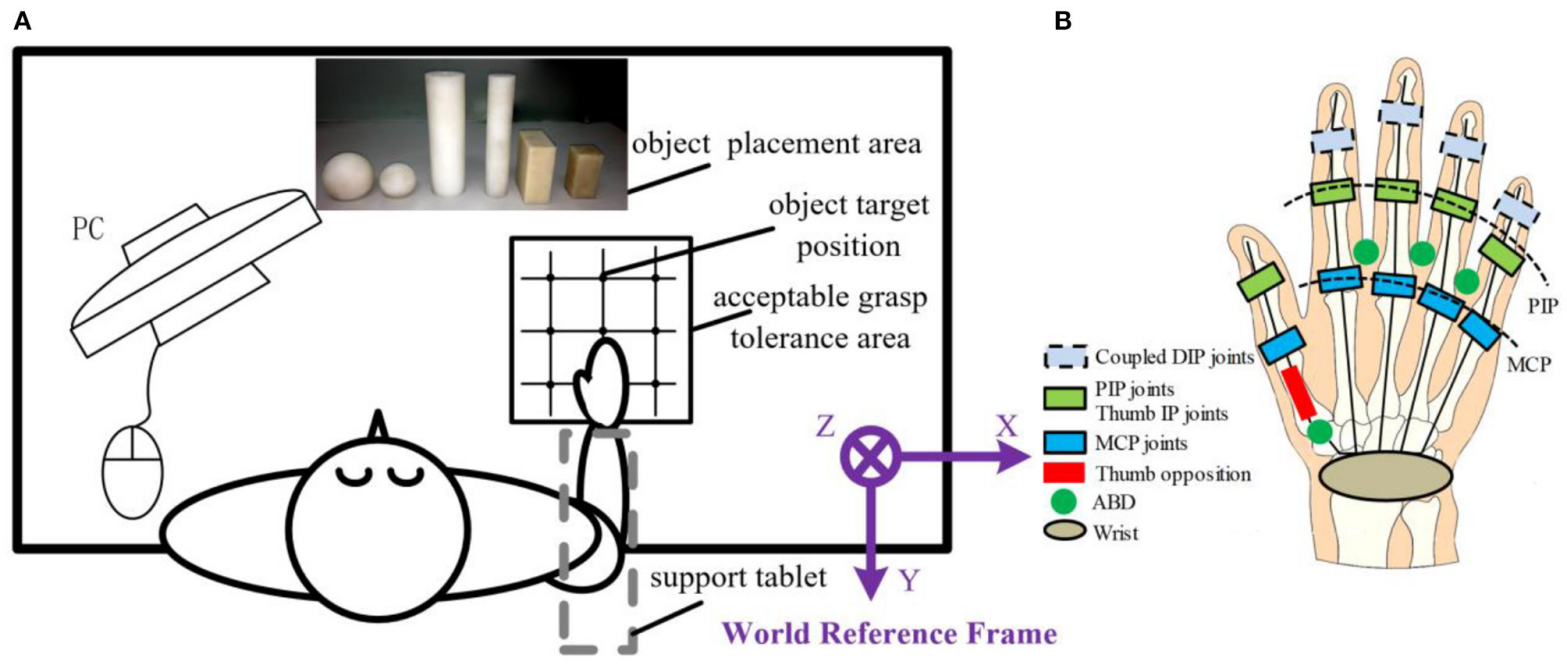
Experimental setup. **(A)** Top view of the experimental setup. **(B)** Schema of the Cyberglove kinematic model contained 15 active joints.

The experimental setup, protocol (contained hand, wrist and arm starting position, experimental paradigm, etc.), data record, and calibration are reported in our recent study (Mason et al., 2001). Ten healthy, right-handed subjects (24–27 years old, eight men and two women) were asked to grasp six objects (three shape × two size) in 27 different relative positions (3 X deviation × 3 Y deviation × 3 Z deviation) between human hand and objects as shown in Figure 7. The object shape, size, and weight (Table 2) were chosen based on the Yale human grasping data set (Feix et al., 2014a,b) for efficiently representing the objects we grasp in daily life. Three typical shapes—sphere, cylinder, and prism—are selected. To embody the effect of object size on grasp, each shape is divided into large and small sizes. Therefore, in total, six objects are selected in our experiment. As 96% of grasp locations are 70 mm or less in width in ADL (Feix et al., 2014a), the smallest sizes of five objects are lower than 70 mm. The large sphere size is larger than 70 mm to cover the large size type. As 92% of objects have a mass of 500 g or less in ADL (Feix et al., 2014a), five objects' weight is lower than 500 g. The large cylinder weight is larger than 500 g to cover the large weight type. As the object prehensile taxonomy shows that the relative position is a significant and important impact factor, we give it particular attention. The 27 different relative positions (3 X deviation × 3 Y deviation × 3 Z deviation) are set in the experiment.

**Table 2:** Shape, size, and weight of the six grasping objects.

**Shape**		**Size (mm)**	**Weight (*g*)**
Sphere	Large	Diameter 80	300
	Small	Diameter 60	100
Cylinder	Large	Diameter 60; height 200	650
	Small	Diameter 40; height 200	300
Prism	Large	Length:80;width:40;height:100	300
	Small	Length:40;width:40;height:100	150

The static grasping postures that can lift up the object are recorded. We hope to obtain a common joint angle feature for the same task rather than the individual cases. Therefore, two repetitions are performed. Thus, in total, 3,240 postures (10 subjects × 6 objects × 27 relative distances × 2 repetitions) are collected. The mean of two repeated trials was used in all statistical analyses. Hand grasp postures containing 15 joint angles were recorded by Cyberglove III (Virtual Technologies, Palo Alto, CA) at a resolution of < 0.1° and sampled at 100 Hz each. The following joint angles were measured: PIP joints and metacarpo-phalangeal (MCP) joints of four fingers except for the thumb as well as the interphalangeal (IP) and MCP joints of the thumb: opposition rotation (Rot) of the thumb, abduction/adduction (ABD) of thumb carpometacarpal (CMC) joint and MCP joints between four fingers except for the thumb.

Principal component analysis (PCA) is used to reduce the dimensionality of hand grasp postures and efficiently represent hand movement characteristics to help clearly understand human grasp behavior influenced by relative position, object shape, and size. In this paper, a five-factor ANOVA (object shape, object size, X deviation, Y deviation, and Z deviation) was performed to investigate the effects on each PC score. Independent factors were object shape (1–3), object size (1–2), X deviation (1–3), Y deviation (1–3), and Z deviation (1–3) between human hand and object. The dependent variables were the first four PC scores.

**Figure 8:**
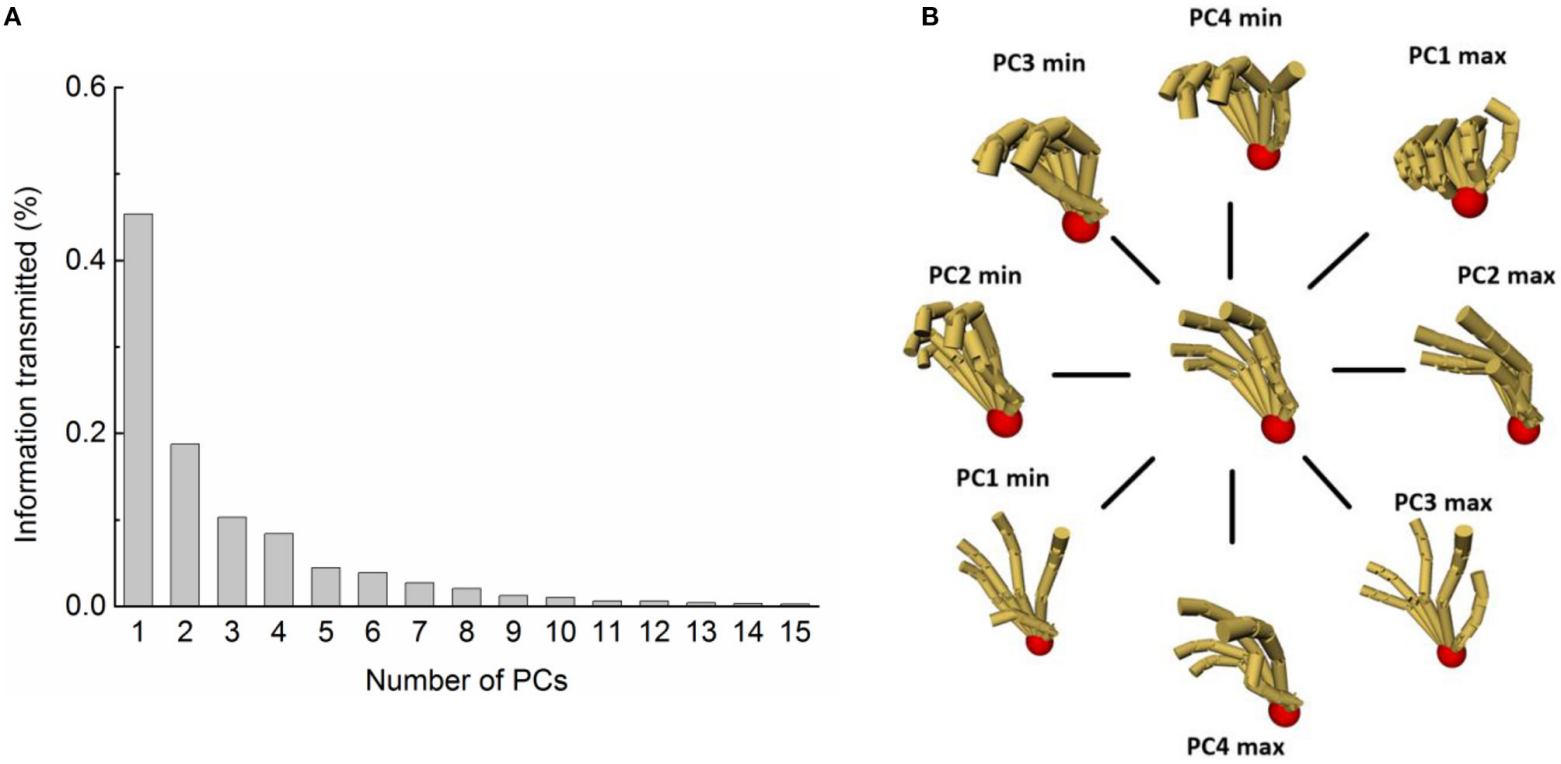
PCA on hand grasp with different shape, size objects in different relative positions. **(A)** Information transmitted by PC1-PC4. **(B)** Hand posture reconstruction by PC1-PC4.

Figure 8A shows the explained variance of each PC. We chose the first four postural synergies (PC1–PC4) to represent hand movement functionality as PC1–PC4 can explain much of total posture variance (82.8%). Figure 8B shows the joint movement characteristics of each PC. The PC1 mainly reflects the major flexion of four-finger MCP and thumb IP joints, major thumb inverse opposition, and minor flexion of four-finger PIP joints. For ADL grasping, this could be characterized by cylinder grasping (such as the pen, cup, handle, mug, et al.) and flat thin object grasping (such as spoon, wrench, card, dinner plate, et al.), accounting for 45.3% of variance. The PC2 explains 18.7% of the variance and was characterized by converse motion between four-finger MCP joints and all PIP joints; this could be used to grasp flat objects (such as book, plate, brick, disk, et al.) in the palm-opposability or pad-opposability posture. PC3 and PC4 explain 10.3 and 8.4% of the variance, respectively. PC3 was characterized by major extension of four-finger MCP and thumb inverse opposition, minor extension of four-finger PIP, ABD of index, ring, and pinky. It shows an opposability grasp of sphere objects (like apple, tennis ball, et al.). The PC4 was mainly characterized by thumb motion containing IP flexion and opposition and the ABD of index finger, ring finger, and pinky.

More importantly, the PCA analysis can estimate the amount of information in grasp postures by comparing the explained variance of postural synergies (PCs). The results show that the grasp postures in our experiment include more information when relative position is considered. The explained variance by the same number of PCs is much less than in other research. The first two PCs of tolerance grasping can only explain <65% (64.1%) of the information. This is obviously lower than other studies of hand kinematic synergies in related studies [~80% in grasp imagined objects (Santello et al., 1998), ~99% in reach-to-grasp for columnar objects (Mason et al., 2001), 70% in biometrics for secure identity verification (Vrajeshri et al., 2017), ~99% in precision grasping for cylinders of different size (Park et al., 2014), ~70% in haptic exploration (Thakur et al., 2008), ~80% in rapid grasping (Mao et al., 2010), ~88% in bimanual manipulation (Jarrassé et al., 2014)]. This indicates that the amount and dimension of information in tolerance grasping is increased with the simultaneous consideration of relative position, object shape, and size. These results quantitatively demonstrate that, after considering the relative position impact on hand grasp, the tolerance grasping and prehensile taxonomy presented here can represent human grasp functionality more comprehensively.

The five-factor ANOVA results are shown in Table 3 for quantifying the factor effects on hand grasping. It can be seen from Table 3 that Y deviation significantly affects PC1, PC2, and PC4. From the *F*-value, it has the most significant effect on PC1. Object size significantly affects PC2–PC4. From the *F*-value, the size has the most significant effect on PC3. For object shape, it significantly affects PC1–PC4, and the impact is more obvious to PC1. For X and Z deviation, the impact is smaller than Y deviation, object shape, and size. They both have the most significant effect on PC1. There were also some significant interactions (from PC1 to PC4, the most significant interaction impact is shape-Y deviation, shape-size-Y deviation, shape-size, and shape-Y deviation, respectively). The Electronic Supplementary Material provides a parameterized understanding of human grasp behavior driven by object shape, size, and relative position in detail.

**Table 3:** *F*-values of the ANOVA on the factor scores of each PC.

	**Size**	**Shape**	**X deviation**	**Y deviation**	**Z deviation**	**Interaction**
PC1		***F***_**(2)**_ **=** **150.1**	***F***_**(2)**_ **=** **97.17**	*F*_(2)_ = **1169.92**	*F*_(2)_ = 6.95	*F*_(4)_ = 25.58
PC2	*F*_(1)_ = 53.2	*F*_(2)_ = 16.72	*F*_(2)_ = 16.48	*F*_(2)_ = 176.7		*F*_(4)_ = 14.49
PC3	*F*_(1)_ = **82.36**	*F*_(2)_ = 58.47			***F***_**(2)**_ **=** **28.22**	*F*_(2)_ = 7.57
PC4	*F*_(1)_ = 59.1	*F*_(2)_ = 49.47	*F*_(2)_ = 33.37	*F*_(2)_ = 8.61		*F*_(4)_ = 9.46

*F_(b)_ = F_(a, b)_, a = 1,522. Only significant factors are shown in the table (p  ≤  0.01). Only the maximum value of F in all interaction factors is shown in the interaction. Bold values indicated most obvious changing PC influenced by each factor*.

Consequently, we mainly observed that (1) Y deviation is the most significant impact factor for PC1 and PC2 due to the maximum value of F in Table 3, PC1 and PC2 performing a three (Supplementary Figure 1) and two ladder-like (Supplementary Figures 2A–C) distribution under the significant impact of Y deviation changing from distal to proximal. (2) Object sizes largely impact the higher order PC (PC2–PC4) as shown in Table 3, Supplementary Figures 2A-C, 3, 4A–C. Different from PC1, higher order PC mainly controls the hand to precisely contact and grasp the object. (3) Object shapes significantly impact PC1–PC4 scores as shown in Table 3. The impact on PC1 is most obvious across PC1–PC4 as shown in Table 3, Supplementary Figure 1. Especially when the object is in the distal and middle Y deviation, PC1 varies across different shapes; the largest is the prism, followed by the cylinder and sphere, which means that the flexion of four-finger MCP joints increases in the order of sphere, cylinder, and prism. (4) The X and Z deviation impact on hand grasping is obviously lower than the Y deviation, object sizes, and shapes as shown in Table 3, Supplementary Figures 1, 2A-C, 3, 4A–C.

## Discussion

This paper provides a systematic framework to help understand hand movement functionality more comprehensively with both qualitative and quantitative analysis. Two novel observations are found: (1) The arm and wrist make a vital contribution to hand movement functionality, and (2) the relative position is a general influence factor significantly impacting human prehension. Finally, a laboratory-based unstructured behavioral experiment is implemented to quantitatively demonstrate the significant influence and understand the human grasp parameterized as driven by object shape, size, and relative positions. The results presented here should be more representative and complete to understand human grasp functionality.

The hierarchical tree presented in section Analysis of Hand Movement Functionality can be used to help decompose the complex task and help understand hand movement skills in ADLs. For the task-contained complex time-separated sequence, the complex task can be decomposed into multiple more specific subtasks according to the hierarchical tree. For instance, drinking hot water might be decomposed into six subtasks: reaching for the cup (class 8), exploring the cup temperature whether hot or not as an antenna (class 7), choosing one distinct grasp type from prehensile taxonomy to grasp the cup stably (class 1) if the cup is not very hot, the arm and wrist transferring the hand to lift the cup to the mouth, and pouring the water into the mouth (class 2), putting the cup back on the table with a stable grasp (class 2), letting the cup, arm, and hand go back to the initial position (class 8), and finally finishing the whole task with a rest position (class 6).

For the bimanual task, both hand movements can be clearly described. For instance, taking pills can be decomposed into 12 subtasks: reaching for the pill bottle with the left hand (class 8), choosing one distinct grasp type from the prehensile taxonomy to grasp the bottle stably by the left hand (class 1), the left arm and wrist transferring the left hand to the front of the chest (class 2), reaching for the bottle cap with the right hand (class 8), both hands keeping a stable grasp and loosening the bottle cap with both arms and wrists helping if the bottle cap is tight enough (class 2), the left hand keeping the stable grasp (class 1) and right hand screwing off the bottle cap by the within-hand rotation about the z-axis from the prehensile taxonomy (class 3), if the bottle cap is loose, putting the bottle cap on the table with the right hand (class 2), the left hand imparting the bottle motion to pour the pills into the right hand (class 2) while the right hand with a platform push type gets the pills (class 5), the left hand putting the bottle on the table (class 2), the right hand putting the pills in the mouth (class 2), and finally finishing the pill-taking task.

The systematic analysis of hand movement functionality also can be used to explore the manner of neuroscience control. Human hands, having more than 24° of freedom (DoF), are actuated by multiple intrinsic and extrinsic muscles by means of a complex web of tendons under the control of the central nervous system. Although extensive investigations have been carried out in biomechanics and the neuroscience of the human hand, there are still many difficulties needing further exploration, such as human hands having how many DoF (especially for the thumb and finger metacarpus), grasp kinematics and impedance properties for different tasks, movement correlations between hand joints under biomechanical constraints, muscle synergies and neural synergies of hand control, brain activity in the grasping process, etc. We believe our systematic analysis of human hand movement functionality can serve this research. Based on the hierarchical tree of hand action and hand prehensile taxonomy, researchers can develop different experimental protocols contributing to their research.

Because the hand prehensile taxonomy presents a descriptive vocabulary of human prehensile behaviors, it can be used to inform design related to the hand, such as product packages (DiSalvo and Gemperle, 2003) on which hands act and assistive devices. As a representative instance of assistive devices, the prosthetic hand is a reconstruction device of human hand functionality (Catalano et al., 2014; Xiong et al., 2016). Amputees wear it to accomplish lots of versatile grasp tasks in their daily lives. Under the limitations of being anthropomorphic, lightweight, robust to uncertainty, and durable, a prosthetic hand should be actuated by a small number of actuators but still have versatile grasp functionality. It is important to build the actuation strategies contributing to the reconstruction of versatile grasp functionality. Based on our proposed prehensile taxonomy, the contribution of each hand DoF to the distinct grasp and manipulative types can be evaluated. In this case, the actuation strategies can be constructed. For reconstructing the move characteristics of the hand joints, the joint angles or positions of each type in prehensile taxonomy can be analyzed and implemented by a mechanism. Moreover, the impedance properties for each grasp type in different tasks can be explored and also can be mechanically implemented by embedding some elastic elements.

The systematic analysis of hand movement functionality also can be used in the clinical rehabilitation and assessment of hand movement capabilities (Light et al., 2002). The hierarchical tree of hand action helps therapists to develop the standardized structure of assessment protocols. The prehensile taxonomy provides a detailed, wide-ranging congregation for developing the protocols on a detailed level. For assistive devices, they also can be used to evaluate and improve the performance contained in mechanical implementation and human–computer interaction.

Even though hand movement functionality has been studied for many years, people are still used to analyze qualitative and quantitative perspectives separately. In this paper, we provide an integral framework to understand human hand movement functions both from qualitative and quantitative perspectives. In qualitative analysis, the general term “hand movement function” is specific to the action class and the detailed postures of prehensile taxonomy. Therefore, we find that the relative position is a significant impact factor except for object shape and size. After simultaneously considering these three impact factors, we quantitatively investigated the posture changing characteristics impacted by these factors through the behavior experiment. Therefore, we hope the qualitative analysis can provide a comprehensive consideration of hand movement functionality and the quantitative analysis can reveal the characteristics of hand movement driven by significant influencing factors. However, several limitations can be identified.

First, the hierarchical tree is a top-level consideration. The aim is to provide a systematic framework about hand movement functionality. However, it does not capture many of the low-level details about hand posture. We pay particular attention to prehensile functionality. The representative postures of two action classes about hold and within-hand manipulation are collected in prehensile taxonomy. The non-prehensile movement might be given additional detailed attention through further subclassification of our hierarchical tree. Second, according to the hierarchical tree, we find that contribution of the wrist and arm for hand movement functionality is important. Although it is easy to accept, the quantitative evidence of arm and wrist contribution should be further investigated, such as the contribution ratio of each hand action, etc. Third, the behavioral experiments considered the significant influencing factors obtained from the analysis of prehensile taxonomy, but the collected postures are not the specific postures of the prehensile taxonomy. It is very interesting that we provide a quantitative evaluation of the posture diversity construction ability to prehensile taxonomy by PCs of behavioral experiment (see Supplementary Material). As the mean construction errors to each posture are 5°-10° (Supplementary Figure 6), the PCs perform diverse posture construction ability. However, the maximum construction error joints to each posture are mainly distributed in the thumb, pinky, and index finger as shown in Supplementary Figure 7. Therefore, we know that the independent movements of these fingers are relatively difficult to accurately construct by the PCs.

## Conclusion

In this paper, we provide a systematic framework to help understand human hand movement functionality from an integral consideration: (1) Hand movement functionality is decomposed and specific to the action as the smallest composition unit of movement functionality. (2) A hierarchical tree is built to classify the action to eight classes based on the hitherto significant definitions of hand movement. (3) A hand prehensile taxonomy containing 52 grasp types is built to provide a macroscopic understanding of hand prehensile function containing both static hold and within-hand manipulations. (4) Prehensile taxonomy is rearranged into object prehensile taxonomies to further explore the general influence factors of hand prehension. (5) Based on the influence factor exploration result, a laboratory-based unstructured behavioral experiment is implemented to comprehensively understand the human grasp and qualitatively demonstrate the significant impact factors.

Consequently, a general analysis method of human movement functionality is presented in this paper and applicable to other body parts, such as wrist, arm, etc. We believe the novel and integral framework of hand movement functionality presented here should help understand hand movement functionality more comprehensively and precisely.

## Data Availability Statement

The raw data supporting the conclusions of this article will be made available by the authors, without undue reservation.

## Ethics Statement

The studies involving human participants were reviewed and approved by Institutional Review Board (IRB) of Harbin Institute of Technology. The patients/participants provided their written informed consent to participate in this study.

## Author's Note

In 2019 YL was awarded the Peiyang Elite Scholar Program of Tianjin University.

## Author Contributions

YL developed the original concept, drafted this paper, and analyzed the resulting data. LJ gave advice on the research methods. HL provided the necessary testing facilities and equipment essential for this research. DM gave some advice on the research methods and helped review the content of the paper. All authors contributed to the article and approved the submitted version.

## Conflict of Interest

The authors declare that the research was conducted in the absence of any commercial or financial relationships that could be construed as a potential conflict of interest.

## References

[B1] Abbasi B. Noohi E. Parastegari S. Zefran M. (2016). Grasp taxonomy based on force distribution, in IEEE International Symposium on Robot & Human Interactive Communication (New York, NY: IEEE), 1098–1103. 10.1109/ROMAN.2016.7745245

[B2] Ansuini C. Giosa L. Turella L. Altoè G. Castiello U. (2008). An object for an action, the same object for other actions: effects on hand shaping. Exp. Brain Res., 185, 111–119. 10.1007/s00221-007-1136-4 17909766

[B3] Bullock I. M. Borràs J. Dollar M. A. (2012). Assessing assumptions in kinematic hand models: a review, in 2012 4th IEEE RAS & EMBS International Conference on Biomedical Robotics and Biomechatronics (BioRob) (IEEE), 139–146. 10.1109/BioRob.2012.6290879

[B4] Bullock I. M. Ma R. Dollar M. A. (2013a). A hand-centric classification of human and robot dexterous manipulation. IEEE Trans. Hapt. 6, 129–144. 10.1109/TOH.2012.53 24808298

[B5] Bullock I. M. Zheng J. Z. Rosa S. D. L. Guertler C. Dollar A. M. (2013b). Grasp frequency and usage in daily household and machine shop tasks. IEEE Trans. Hapt. 6, 296–308. 10.1109/TOH.2013.6 24808326

[B6] Cartmill M. (1974). Rethinking primate origins. Science 184, 436–443. 10.1126/science.184.4135.436 4819676

[B7] Castiello U. (2005). The neuroscience of grasping. Nat. Rev. Neurosci. 6, 726–736. 10.1038/nrn1744 16100518

[B8] Catalano M. G. Grioli G. Farnioli E. Serio A. Piazza C. Bicchi A. (2014). Adaptive synergies for the design and control of the Pisa/IIT SoftHand. Int. J. Robot. Res. 33, 768–782. 10.1177/0278364913518998

[B9] Cohen G. R. Rosenbaum A. D. (2004). Where grasps are made reveals how grasps are planned: generation and recall of motor plans. Exp. Brain Res. 157, 486–495. 10.1007/s00221-004-1862-9 15071711

[B10] Cutkosky R. M. (1989). On grasp choice, grasp models, and the design of hands for manufacturing tasks. IEEE Trans. Robot. Automat. 5, 269–279. 10.1109/70.34763

[B11] DiSalvo C. Gemperle F. (2003). From seduction to fulfillment: the use of anthropomorphic form in design, in Proceedings of the 2003 International Conference on Designing Pleasurable Products and Interfaces (Pittsburgh, PA), 67–72. 10.1145/782896.782913

[B12] Elliott M. J. Connolly K. (1984). A classification of manipulative hand movements. Dev. Med. Child Neurol. 26, 283–296. 10.1111/j.1469-8749.1984.tb04445.x 6734945

[B13] Feix T. Bullock I. Gloumakov Y. Dollar A. (2020). Effect of number of digits on human precision manipulation workspaces. IEEE Trans. Hapt. 2020:1. 10.1109/TOH.2020.3003556 32746375

[B14] Feix T. Bullock I. M. Dollar M. A. (2014a). Analysis of human grasping behavior: object characteristics and grasp type. IEEE Trans. Hapt. 7, 311–323. 10.1109/TOH.2014.2326871 25248214

[B15] Feix T. Bullock I. M. Dollar M. A. (2014b). Analysis of human grasping behavior: correlating tasks, objects and grasps. IEEE Trans. Hapt. 7, 430–441. 10.1109/TOH.2014.2326867 25532148

[B16] Feix T. Romero J. Ek C. H. Schmiedmayer H-B. Kragic D. (2012). A metric for comparing the anthropomorphic motion capability of artificial hands. IEEE Trans. Robot. 29, 82–93. 10.1109/TRO.2012.2217675

[B17] Feix T. Romero J. Schmiedmayer H.-B. Dollar A. M. Kragic D. (2015). The grasp taxonomy of human grasp types. IEEE Trans. Hum. Machine Syst. 46, 66–77. 10.1109/THMS.2015.2470657

[B18] Goodale A. M. (2010). Transforming vision into action. Vis. Res. 51, 1567–1587. 10.1016/j.visres.2010.07.027 20691202

[B19] Iberall T. (1986). Opposition space as a structuring concept for the analysis of skilled hand movements. Generat. Modulat. Action Patterns 15, 158–173. 10.1007/978-3-642-71476-4_12

[B20] Iberall T. (1987). Grasp planning from human prehension. IJCAI 87, 1153–1157

[B21] Iberall T. (1997). Human prehension and dexterous robot hands. Int. J. Robot. Res. 16, 285–299. 10.1177/027836499701600302

[B22] Jakobson L. Goodale A. M. (1991). Factors affecting higher-order movement planning: a kinematic analysis of human prehension. Exp. Brain Res. 86, 199–208. 10.1007/BF00231054 1756790

[B23] Jarrassé N. Ribeiro A. T. Sahbani A. Bachta W. Roby-Brami A. (2014). Analysis of hand synergies in healthy subjects during bimanual manipulation of various objects. J. NeuroEng. Rehabil. 11:113. 10.1186/1743-0003-11-113 25077840 PMC4237861

[B24] Juravle G. Deubel H. Spence C. (2011). Attention and suppression affect tactile perception in reach-to-grasp movements. Acta Psychol. 138, 302–310. 10.1016/j.actpsy.2011.08.001 21872190

[B25] Kamakura N. Matsuo M. Ishii H. Mitsuboshi F. Miura Y. (1980). Patterns of static prehension in normal hands. Am. J. Occup. Therapy 34, 437–445. 10.5014/ajot.34.7.437 6446851

[B26] Kamper D. G. Cruz E. G. Siegel P. M. (2003). Stereotypical fingertip trajectories during grasp. J. Neurophysiol. 90, 3702–3710. 10.1152/jn.00546.2003 12954607

[B27] Kapandji I. (1971). The physiology of the joints, volume I, upper limb. Am. J. Phys. Med. Rehabil. 50:96.

[B28] Light C. M. Chappell P. H. Kyberd J. P. (2002). Establishing a standardized clinical assessment tool of pathologic and prosthetic hand function: normative data, reliability, and validity. Archiv. Phys. Med. Rehabil. 83, 776–783. 10.1053/apmr.2002.32737 12048655

[B29] Loclair C. Gustafson S. Baudisch P. (2010). PinchWatch: a wearable device for one-handed microinteractions. Proc. MobileHCI, 2010:10.

[B30] Mao Z. Sclabassi R. J. Lee H. Chang C. C. Sun M. Vinjamuri R. (2010). Temporal postural synergies of the hand in rapid grasping tasks. IEEE Trans. Inform. Technol. Biomed. 14, 986–994. 10.1109/TITB.2009.2038907 20071263

[B31] Mason C. R. Gomez J. E. Ebner J. T. (2001). Hand synergies during reach-to-grasp. J. Neurophysiol. 86, 2896–2910. 10.1152/jn.2001.86.6.2896 11731546

[B32] Meier J. D. Aflalo T. N. Kastner S. Graziano S. M. (2008). Complex organization of human primary motor cortex: a high-resolution fMRI study. J. Neurophysiol. 100, 1800–1812. 10.1152/jn.90531.2008 18684903 PMC2576195

[B33] Nakamura Y. C. Troniak D. M. Rodriguez A. Mason M. T. Pollard S. N. (2017). The complexities of grasping in the wild, in 2017 IEEE-RAS 17th International Conference on Humanoid Robotics (Humanoids) (Birmingham), 233–240. 10.1109/HUMANOIDS.2017.8246880

[B34] Napier R. J. (1956). The prehensile movements of the human hand. J. Bone Joint Surg. 38, 902–913. 10.1302/0301-620X.38B4.902 13376678

[B35] Park J. Seo N. J. Son J. Kim W. Cheong J. (2014). Postural variation of hand precision grips by object size. J. Mechanical. Technol. 28, 1641–1651. 10.1007/s12206-014-0309-x

[B36] Preuschoft H. Chivers D. J. (2012). Hands of Primates. New York, NY: Springer Science & Business Media.

[B37] Rosenbaum D. A. van Heugten M. C. Caldwell E. G. (1996). From cognition to biomechanics and back: the end-state comfort effect and the middle-is-faster effect. Acta Psychol. 94, 59–85. 10.1016/0001-6918(95)00062-3 8885711

[B38] Sanes J. N. Donoghue J. P. Thangaraj V. Edelman R. R. Warach S. (1995). Shared neural substrates controlling hand movements in human motor cortex. Science 268, 1775–1777. 10.1126/science.7792606 7792606

[B39] Santello M. Baud-Bovy G. Jörntell H. (2013). Neural bases of hand synergies. Front. Computat. Neurosci. 7:23. 10.3389/fncom.2013.00023 PMC3619124 23579545

[B40] Santello M. Flanders M. Soechting F. J. (1998). Postural hand synergies for tool use. J. Neurosci. 18, 10105–10115. 10.1523/JNEUROSCI.18-23-10105.1998 9822764 PMC6793309

[B41] Schieber H. M. Hibbard S. L. (1993). How somatotopic is the motor cortex hand area? Science 261, 489–492. 10.1126/science.8332915 8332915

[B42] Schieber H. M. Santello M. (2004). Hand function: peripheral and central constraints on performance. J. Appl. Physiol. 96, 2293–300. 10.1152/japplphysiol.01063.2003 15133016

[B43] Schlesinger G. (1919). Der mechanische aufbau der künstlichen glieder. (Ersatzglieder und Arbeitshilfen: Springer), 321–661. 10.1007/978-3-662-33009-8_13

[B44] Sereno B. A. Maunsell H. J. (1998). Shape selectivity in primate lateral intraparietal cortex. Nature 395, 500–503. 10.1038/26752 9774105

[B45] Smeets B. J. Brenner E. (1999). A new view on grasping. Motor Control 3, 237–271. 10.1123/mcj.3.3.237 10409797

[B46] Stillfried G. van der Smagt P. (2010). Movement Model of a Human Hand Based on Magnetic Resonance Imaging (MRI) (Stillfried: International Federation of Medical and Biological Engineering; IEEE Engineering in Medicine and Biology Society; IEEE Robotics and Automation Society).

[B47] Tessitore G. Sinigaglia C. Prevete R. (2013). Hierarchical and multiple hand action representation using temporal postural synergies. Exp. Brain Res. 225, 11–36. 10.1007/s00221-012-3344-9 23229775

[B48] Thakur P. H. Bastian A. J. Hsiao S. S. (2008). Multidigit movement synergies of the human hand in an unconstrained haptic exploration task. J. Neurosci. Off. J. Soc. Neurosci. 28, 1271–1281. 10.1523/JNEUROSCI.4512-07.2008 18256247 PMC6671569

[B49] Touvet F. Roby-Brami A. Maier M. A. Eskiizmirliler S. (2014). Grasp: combined contribution of object properties and task constraints on hand and finger posture. Exp. Brain Res. 232, 3055–3067. 10.1007/s00221-014-3990-1 24888535

[B50] Vrajeshri P. Poojita T. Burns M. K. Ionut F. Rajarathnam C. Ramana V. (2017). Hand grasping synergies as biometrics. Front. Bioeng. Biotechnol. 5:26. 10.3389/fbioe.2017.00026 28512630 PMC5411425

[B51] Wolpert M. D. Ghahramani Z. (2000). Computational principles of movement neuroscience. Nat. Neurosci. 3, 1212–1217. 10.1038/81497 11127840

[B52] Xiong C.-H. Chen W.-R. Sun B.-Y. Liu M.-J. Yue S.-G. Chen W.-B. (2016). Design and implementation of an anthropomorphic hand for replicating human grasping functions. IEEE Trans. Robot. 32, 652–671. 10.1109/TRO.2016.2558193

[B53] Zhan Q. Liu X. (2013). Hand grasp function analysis based on VF set. Int. J. Humanoid Robot. 10:1350026. 10.1142/S0219843613500266

