# Supplementary material for: A Systematic Analysis of Hand Movement Functionality: Qualitative Classification and Quantitative Investigation of Hand Grasp Behavior
Source: Front Neurorobot. 2021 Jun 7;15:658075. doi: 10.3389/fnbot.2021.658075 (PMC8216684; doi:10.3389/fnbot.2021.658075)
Supplement: Supplementary file 1 [file Data_Sheet_1.PDF]

Frontiers in Neurorobotics

A Systematic Analysis of Hand Movement Functionality: Qualitative Classification and Quantitative Investigation of Hand Grasp Behavior

(*Electronic supplementary material*)

Yuan Liu[1], Li Jiang[2], Hong Liu[2], Dong Ming[1]

[1]Academy of Medical Engineering and Translational Medicine
Tianjin University
Tianjin, 300072, China.

[2]State Key Laboratory of Robotics and System,
Harbin Institute of Technology,
Harbin, Heilongjiang 150080, China.

This document includes:

- Supplementary explanation to the selection of the manipulative postures in our taxonomy

-Supplementary table (S1_table)

- Supplementary explanation to PC1-PC4 scores parameterizedly driven by object shape, size and relative positions

- Supplementary figure (S1_fig- S4_fig)

- Supplementary explanation to the posture diversity construction ability by PCs

- Supplementary figure (S5_fig- S7_fig)

1. The explanation to the selection of the manipulative postures in our taxonomy

We selected two highly cited papers on the classification of hand manipulation behavior [33-34] to help efficiently summarize within hand manipulation ability in our taxonomy. These two papers classified hand manipulation behavior from the view of the manipulative patterns and object motion with respect to the hand coordinate frame respectively. We unified these two views and arranged them in our prehensile taxonomy. Therefore, manipulation behaviors classified in these two representative papers [33-34] are all covered and presented by finger gaits in our taxonomy.

At first, to the best of our knowledge, [33] first clearly clarify hand manipulation ability and provided a good foundation in related areas. The paper has been cited 139 times in the web of science database. Human manipulative movement is systematically classified into three general classes according to the manipulative patterns, including simple synergies, reciprocal synergies, and sequential patterns. Secondly, [34] presents the classification based on the direction of object motion with respect to the hand. Therefore, three rotational (θX/Y/Z) and three translational (ΔX/Y/Z) movements with respect to the hand coordinate frame are included. However, dexterous motions in a few directions are difficult for the human hand to accomplish given its kinematic abilities (e.g., x-axis translation (ΔX)). Therefore, we arrange the manipulative patterns [33] and object motion with respect to the hand coordinate frame [34] into one same single table. Each manipulative pattern in [33] is picked to correspond to the object motion with respect to human hand, as shown in S1_table.

The terms of S1_table is from [33] and clarified as follows:

1) **Simple synergies:** All movements of the participating fingers, including thumb, are convergent flexor synergies. Three specific patterns are shown in S1_table, and described as follows:
   a) **Pinch:** Concurrent flexion of thumb (opposed position) and index finger moves the object towards palm (ΔZ pinch); Concurrent extension of thumb (adduction position) and index finger moves the object away from palm.
   b) **Dynamic Tripod:** Simultaneous flexion movements of thumb, index and middle finger.
   c) **Squeeze:** With the thumb opposed and all the fingers relatively extended, the object is squeezed by synergistic flexion of the fingers.

2) **Reciprocal synergies:** By contrast, the thumb and the other participating fingers show dissimilar or reciprocating movements, such as flexion of the fingers with adduction or extension of the thumb. Three specific patterns are shown in S1_table, and described as follows:
   a) **Twiddle:** Thumb in partial adduction with concurrent index flexion (θY twiddle), and thumb in partial extension with concurrent index flexion (θZ twiddle). This pattern is used to roll small objects to and fro between thumb and index finger.
   b) **Index Roll:** Slight reciprocal flexion of index finger and extension of thumb.
   c) **Full Roll:** Similar with index roll, but with involvement of additional fingers.

3) **Sequential patterns:** This pattern entails an intermittent rotary movement of an object. The specific patterns are shown in S1_table, and described as follows:
   a) **Rotary step:** The object is stepped around, with brief pauses while the position of the digits is readjusted, using a sequential or phased set of movements.

For the ΔY Pinch and θX Full Roll in S1_table, the manipulation patterns need the synergy motions between the thumb and the other four fingers. The motion range is relatively less than the other patterns in S1_table. Therefore, we use two sequential gait postures to represent the corresponding manipulative pattern. While for other manipulation patterns with larger motion range, we use three sequential gait postures to represent the pattern.

S1_table Human within hand manipulation classification [33-34]

General Class of Movement [33]	Specific Movement Pattern [33]	Principal Axis of Motion [34]	Finger Gaits [33]
Simple Synergies	Pinch	ΔZ	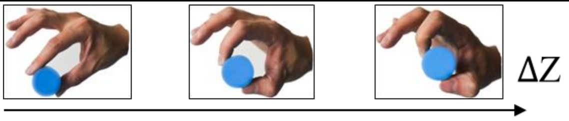
		ΔY	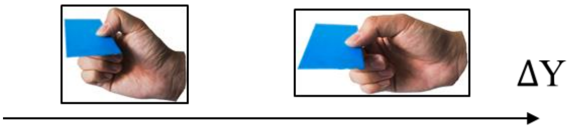
	Dynamic Tripod	ΔZ	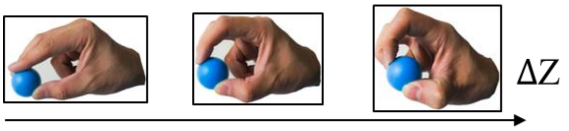
	Squeeze	ΔY	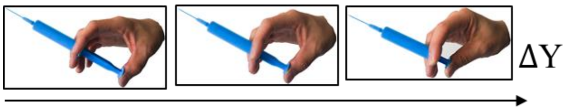
Reciprocal Synergies	Twiddle	θY	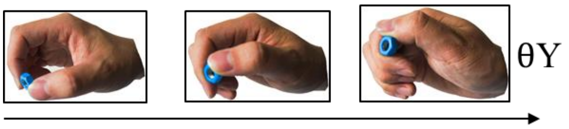
		θZ	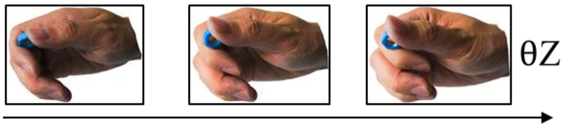
	Index Roll	θX	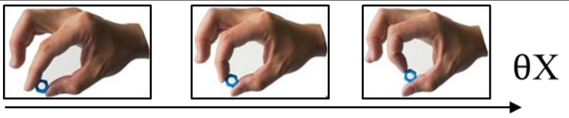
	Full Roll	θX	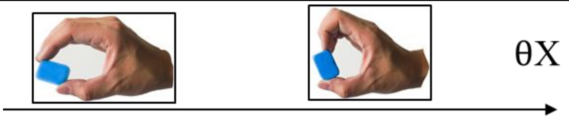
Sequential Patterns	Rotary step	θZ	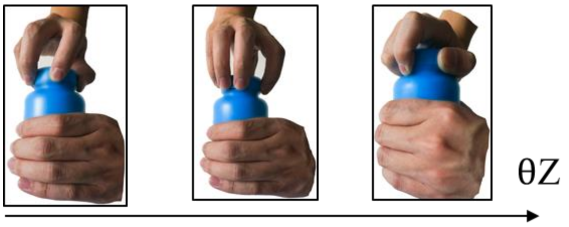

In addition, in order to present a representative prehensile taxonomy, we have chosen the more representative dexterous manipulation patterns. Some limited manipulation tasks are not contained, such as Precision 2-4, 2-5 along the z-direction. The recent study [S1_REF] verified that the extra fingers participated in manipulation will lead to the additional kinematic constraints. The manipulation ability will be significantly reduced. Therefore, except for the Sequential Pattern in S1_table, the other manipulation patterns in prehensile taxonomy are two-finger and three-finger

contact patterns. Moreover, considering the completeness, the selected postures can cover five Principal Axis Motions including ΔZ and θZ, ΔY and θY, and θX. The absence of ΔX is mainly due to the difficulty of the hand in imparting object motion in this direction [34].

[33]. J. M. Elliott and K. Connolly, "A classification of manipulative hand movements," Developmental Medicine & Child Neurology, vol. 26, no. 3, pp. 283-296, 1984.

[34] Bullock, Ian M , R. R. Ma , and A. M. Dollar . "A Hand-Centric Classification of Human and Robot Dexterous Manipulation," IEEE Transactions on Haptics, vol. 6, no. 2, pp.129-144, 2013.

[S1_REF] Bullock, I. M. , T. Feix , and A. M. Dollar . "Human precision manipulation workspace: Effects of object size and number of fingers used." 37th Annual International Conference of the IEEE Engineering in Medicine and Biology Society (EMBC), IEEE, 2015: 5768-5772.

2. The explanation to PC1-PC4 scores parameterizedly driven by object shape, size and relative positions

Consequently, PC scores of S1_fig-S4_fig quantitatively reflect the factor effect result of Table 3. From S1_fig-S4_fig and Table 3, we mainly observed that: (1) Y deviation is the most significant impact factor for PC1 and PC2. As shown in S1_fig and S2_fig (a-c), PC1 and PC2 perform a three (S1_fig) and two ladder-like (S2_fig (a-c)) distribution, under the significant impact of Y deviation changing from distal to proximal; (2) Object sizes largely impact the higher-order PC (PC2~PC4). It can be shown in S2_fig (a-c), S3_fig and S4_fig (a-c). Different with PC1, higher-order PC mainly control hand to precisely contact and grasp the object; (3) Object shapes significantly impact PC1~PC4 scores. The impacts on PC1 is most obvious across PC1~PC4, as shown in Table 3 and S1_fig. Especially when the object is in the distal and middle Y deviation, PC1 varies across different shapes, the largest is the prism, followed by the cylinder and sphere, which means that the flexion of four-finger MCP joints increases in the order of sphere, cylinder and prism; (4) X and Z deviation impact on hand grasping is obviously lower than Y deviation, object sizes and shapes, as shown in Table 3 and S1_fig-S4_fig.

In terms of PC1, S1_fig obviously reflects the ANOVA results, showing that PC1 is influenced significantly by Y/X/Z deviations, object shape and size. Especially for the Y deviation influence, S1_fig shows that the PC1 obviously varies in a three ladder-like distribution under the influence of Y deviation from distal to proximal, which is consistent with the ANOVA results (Table 3) that the most significant influence factor for PC1 is Y deviation. PC1 mainly reflects the large ratio flexion in four-finger MCP joints and thumb IP joint, inverse opposition motion of thumb opposition rotation, small ratio flexion of four-finger PIP joints. PC1 scores increases from negative (extension) to positive (flexion) along with the object Y deviation from distal to proximal based on the mean posture. The results indicate that when the object is in distal position, the four-finger MCP joints extend to touch and grasp the object as much as possible. While when the object is in the proximal, the MCP joints of four-finger flex to stably grasp the object with a power grasp. For object property influence, we can clearly see the PC1 is obviously varied with the object shapes. Especially when the object is in the distal and middle Y deviation, PC1 varies across different shapes, the largest is the prism, followed by the cylinder and sphere, which means that the flexion of four-finger MCP joints increases in the order of sphere, cylinder and prism.

In terms of PC2, due to the relative smaller influence of object shape, our results are presented in three figures (S2_fig) divided by object shapes in order to clearly show the results. Table 3 shows that the Z deviation is not the significant influence factor to PC2. Thus, we abandon the Z deviation interference in S2_fig. S2_fig reflects the ANOVA results (Table 3) obviously. PC2 is influenced significantly by Y (distal-middle-proximal)/X(L-M-R) deviations and object size (Large-Small), shape(Sphere/Cylinder/Prism). For position deviation influence, S2_fig (a-c) shows that PC2 obviously varies in a two ladder-like distribution under the influence of Y deviation from distal to proximal. PC2 mainly controls the larger PIP flexion, smaller MCP extension and thumb IP flexion. When the object is in distal position, PIP joints extend while MCP joint flex based on the mean posture to approach and grasp it with a precision grasp. In addition, for sphere and cylinder object, it is obvious that the effect of object size on PC2 is significant only when the object is located at a relatively proximal position (middle and proximal deviation in S2_fig (a-b). The small size corresponds to smaller PC2. This indicates that when the object is located at a relatively proximal position, PC2 plays an important role to adapt the object size.

In terms of PC3, due to the lower variance explained percentage (10.3%) than PC1 (45.4%) and PC2 (18.7%), the distribution variance along PC3 is far below than that along PC1 and PC2. Meanwhile, the X/Y deviation is not the significant impact factor of PC3 (Table 3). Therefore, we eliminate the interference of X/Y deviation, as shown in S3_fig. The PC3 mainly reflects the larger extension of index-middle-ring finger MCP joints, thumb inverse-opposition rotation, smaller extension of four-finger PIP joints, and abduction index, ring and pinky, as shown in Fig. 8(b). It can be seen from S3_fig that PC3 varies across object size, shape and Z deviations, especially object sizes. The small size corresponds to smaller PC3, which means larger finger flexion.

In terms of PC4, object size, shape, and X (L-M-R)/Y (distal-middle-proximal) deviations. Z (Low-Middle-High) deviation is not the significant impact factor (Table 3) and the lower variance explained percentage (8.4%). We eliminate the

interference of Z(L-M-H) deviation, three figures (S4_fig (a-c)) are provided to show the results clearly. PC4 mainly controls thumb IP flexion and opposition rotation, abduction between five fingers. S4_fig (a-c) results is consistent with the ANOVA result that object size, shape and X(L-M-R)/Y(distal-middle-proximal) deviations. Generally, similar to PC2 and PC3, the small size corresponds to smaller PC3, which means larger finger flexion. In addition, compared with grasping sphere, the impacts of Y deviation is more obvious to grasp cylinder and prism objects. In terms of X deviation impacts, large prism grasping is influenced more significantly than sphere and cylinder objects.

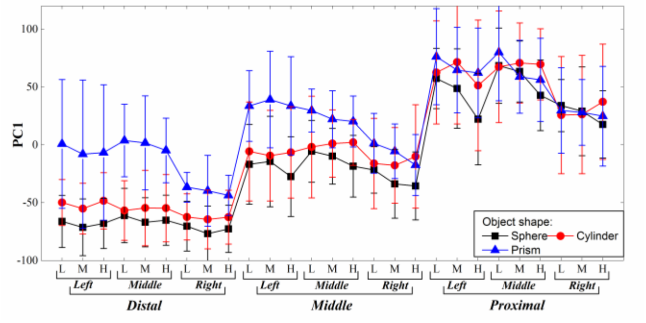

S1_fig. PC1 scores influenced by Y/X/Z deviations, object shape (subject mean and SE).

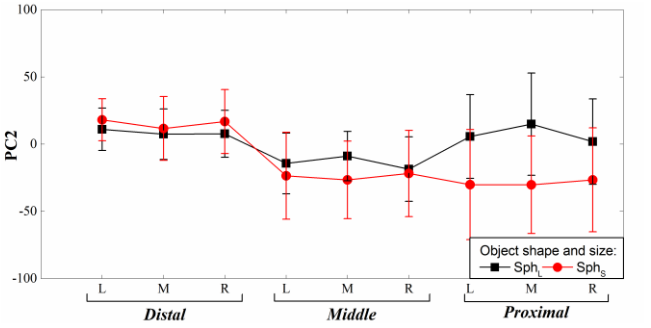

S2_fig (a). PC2 scores of grasping sphere objects influenced by object size and X/Y deviations (subject mean and SE).

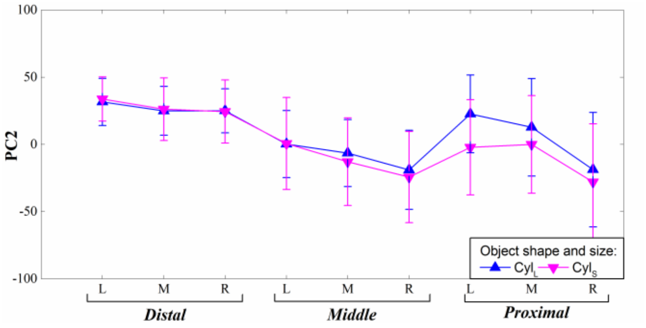

S2_fig (b) PC2 scores of grasping cylinder objects influenced by object size and X/Y deviations (subject mean and SE).

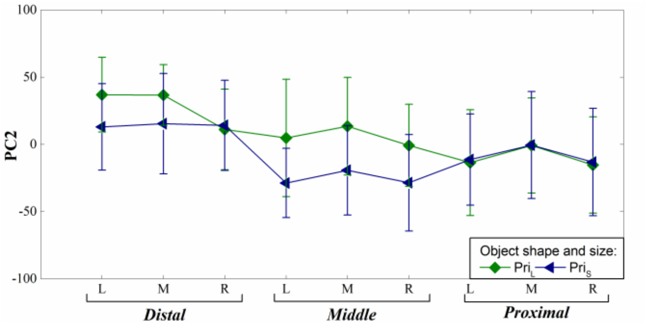

S2_fig (c) PC2 scores of grasping prism objects influenced by object size and X/Y deviations (subject mean and SE).

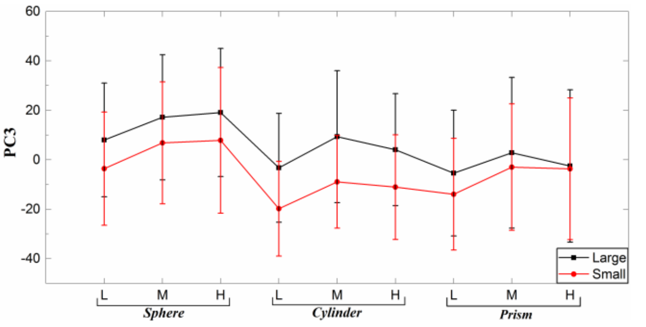

S3_fig. PC3 scores influenced by object shape, size and Z deviations (subject mean and SE).

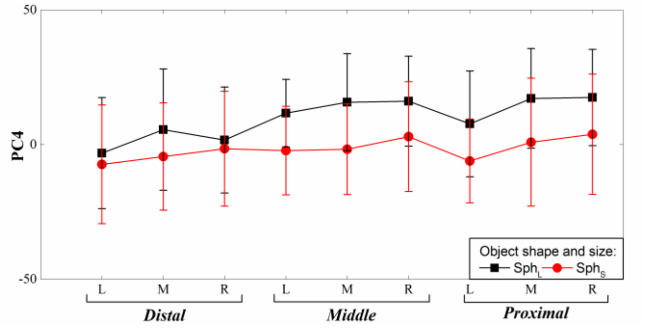

S4_fig (a) PC4 scores of grasping sphere objects influenced by object size and X/Y deviations (subject mean and SE).

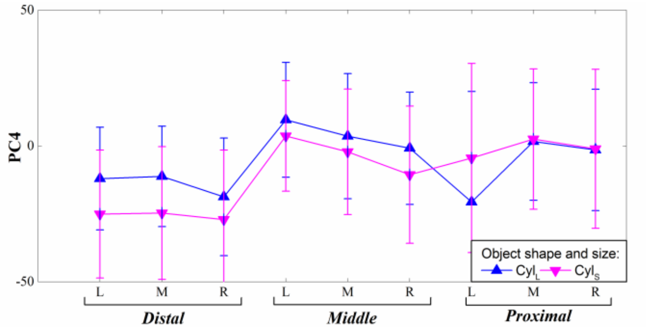

S4_fig (b) PC4 scores of grasping cylinder objects influenced by object

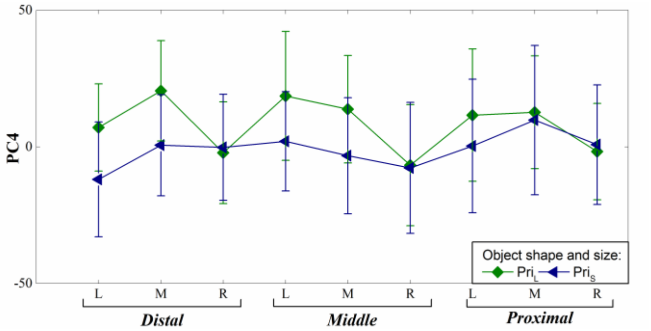

S4_fig (c) PC4 scores of grasping prism objects influenced by object size

3. The explanation to the posture diversity construction ability by PCs of this paper experiment

Feix GRASP taxonomy is used to investigate the posture diversity construction ability by PCs of this paper experiment. The experiment involved one participant who is most familiar with grasp taxonomy among 10 subjects. Referring to the posture in Feix GRASP taxonomy, the subject reproduced the corresponding posture and the posture was recorded by Cyberglove III, as shown in S_fig. 5. Each posture was performed twice. The Cyberglove calibration is the same with the paper. Totally 66 trials (33 postures × 2 repeats) are accomplished. Then, the two repeats were averaged. Thus, HPD-Feix (human posture dataset-Feix grasp taxonomy) is constructed with 33 postures for evaluating the posture diversity construction ability by PCs of this paper experiment. We use four PCs to construct HPD-Feix. The constructed error of the j-th posture in HPD-Feix is calculated:

$$E_j = \frac{\sum_{k=1}^{n} \left| q_{jk}^{HPD-RC} - q_{jk}^{HPD-\mathrm{Feix}} \right|}{n} \qquad \text{(S1\_ equation)}$$

The n represents the number of joints to each recorded posture. The q_{ik}^{HPD-RC} represents the constructed joint angle by PCs, while $q_{jk}^{HPD-\mathrm{Feix}}$ represents the recorded joint angle of Feix GRASP taxomomy. The *j* is the *j*-th posture of HPD-Feix, and *k* is the *k*-th joint. Finally, the mean constructed error of each posture in Feix GRASP taxonomy is shown in S6_fig. It can be seen from the figure that most of the mean construction errors are 5°-10°. The results mean that the PCs of this paper experiment perform diverse posture construction ability. The postures with a more than 10° construction error are mainly distributed in the cells of index finger VF (VF: 2) in S6_fig. Meanwhile, the maximum construction error joints to each HPD-Feix posture are mainly distributed in the thumb, pinky and index finger, as shown in S7_fig. Therefore, we know that the independent movements of these fingers are relatively difficult to be accurately constructed by the PCs.

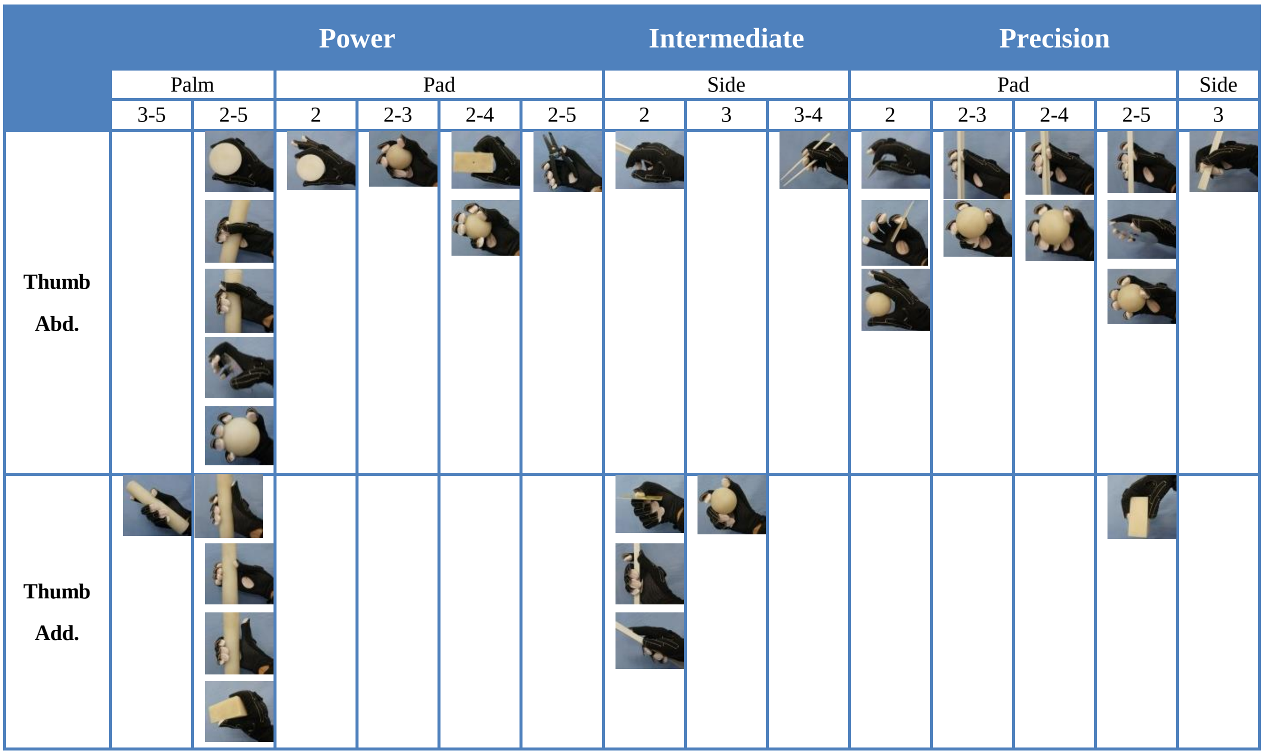

S5_fig. The grasp posture collection protocol of Feix grasp taxonomy

	Power						Intermediate			Precision				
**Opposition:**	Palm		Pad				Side			Pad				Side
**VF:**	3-5	2-5	2	2-3	2-4	2-5	2	3	3-4	2	2-3	2-4	2-5	3
Thumb Abd. (Reconstruction Error) (deg)		6.9	15.6	9	7.3	6.7	12.1		4.2	7.9	5.8	4.4	7.5	4.3
		4			7.8					10.2	7.8	4.6	10	
		4.6								12.7			4.9	
		7.2												
		6.7												
Thumb Add. (Reconstruction Error) (deg)	15.6	6.5					4.2	9.2					9.2	
		8.1					14.3							
		7.1					18.5							
		10.6												

S6_fig. Mean construction error to each HPD-Feix posture

	Power						Intermediate			Precision				
**Opposition:**	Palm		Pad				Side			Pad				Side
**VF:**	3-5	2-5	2	2-3	2-4	2-5	2	3	3-4	2	2-3	2-4	2-5	3
Thumb Abd. (Maximum construction error joint)		P-MCP	P_PIP	ABD-M_P	I_PIP	M_PIP	ABD-M_P		T_MCP	T_MCP	I_PIP	P_PIP	T_MCP	I_PIP
		P-PIP			P_MCP					P_PIP	ABD-I_M	P_PIP	P_MCP	
		I-PIP								P_MCP			T_MCP	
		T-MCP												
		ABD-R_P												
Thumb Add. (Maximum construction error joint)	I-PIP	T-MCP					ABD-I_M	ABD-I_M					T_MCP	
		T-MCP					P_MCP							
		T-MCP					I_PIP							
		T-MCP												

S7_fig. Maximum construction error joint to each HPD-Feix posture. The red, yellow and green background mean the joint of index finger, pinky and thumb, respectively.
